# Green synthesis and characterization of Ag and ZnO nanoparticles and Ag@ZnO nanocomposites using *Taxus wallichiana*: comparative assessment of therapeutic potential and biocompatibility

**DOI:** 10.1039/d6ra04018g

**Published:** 2026-07-03

**Authors:** Mehreen Sarfraz, Shahid Sultan, Amjid Khan, Umer Rehman, Shanzay Saleem, Muhammad Ali, Hamza Elsayed Ahmed Mohamed, Zabta Khan Shinwari

**Affiliations:** a Department of Plant Sciences, Faculty of Biological Sciences, Quaid-i-Azam University Islamabad, 45320 Pakistan shinwari@qau.edu.pk; b UNESCO-UNISA Africa Chair in Nanosciences and Nanotechnologies, College of Graduate Studies, University of South Africa 1 Preller Street, Muckleneuk Ridge, P.O. Box 392 Pretoria Gauteng Province 0003 South Africa khana2@unisa.ac.za; c African Centre of Competencies in Enhanced Nanosciences & Nanotechnologies for SDGs (ACCENTS) 1 Preller Street, Muckleneuk Ridge, P.O. Box 392 Pretoria Gauteng Province 0003 South Africa; d Department of Botany, The University of Punjab Lahore 54590 Pakistan; e Department of Biotechnology, Faculty of Biological Sciences, Quaid-i-Azam University Islamabad 45320 Pakistan; f Dr. A. Q. Khan Institute of Materials & Emerging Sciences – DAKIMES, Quaid-i-Azam University Islamabad 45320 Pakistan; g Physics Department, Institut des Molécules et Matériaux du Mans, Le Mans Université Le Mans France; h Federal Urdu University of Arts, Sciences and Technologies (FUUAST) Karachi 75300 Pakistan

## Abstract

This study reports the first phytochemical-mediated synthesis of AgNPs, ZnONPs, and bimetallic Ag@ZnO nanocomposites (NCs) using leaf extracts of the ethnopharmacologically significant *Taxus wallichiana*. Comprehensive structural profiling (FTIR, XRD, SEM, TEM, and SAED) confirmed that the plant's secondary metabolites successfully act as primary reducing and capping agents, restricting individual primary physical particle diameters to fine nanometer scales (4.94 nm for AgNPs, 15.64 nm for ZnONPs, and 6.42 nm for Ag@ZnO NCs) *via* ImageJ tracking, while driving their assembly into highly textured macro-clusters due to low surface charges underneath the 40 mV threshold (zeta potentials ranging from −16.8 mV to −23.0 mV). These structural arrangements directly correspond to solution-state DLS hydrodynamic sizes of 422.8 nm, 377.2 nm, and 195.6 nm, respectively, confirming that the bimetallic configuration provides significantly improved charge-based stabilization. Due to this unique structural engineering, the Ag@ZnO NCs exhibited potent antioxidant activity (DPPH IC_50_: 340 µg mL^−1^), significant alpha-amylase inhibition (76.8%), and remarkable antibacterial efficacy against *Klebsiella pneumoniae* (38 mm zone). Crucially, biocompatibility assays using human RBCs and brine shrimp larvae confirmed low eco-toxicity and environmental safety. This work highlights a sustainable green chemistry route for developing high-performance, plant-passivated bimetallic nanostructures tailored for advanced biomedical and environmental remediation applications.

## Abstract 2

Biocompatible AgNPs, ZnONPs and Ag@ZnO nanocomposites green-synthesized *via Taxus wallichiana* leaf extract exhibit remarkable multi-therapeutic applications, including robust antioxidant, alpha-amylase inhibitory, and antibacterial activities.

## Graphical abstract

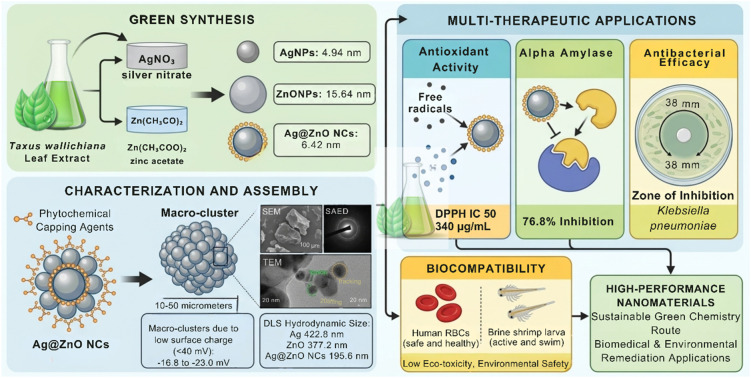

## 1 Introduction

Nanotechnology has become a revolution in biomedical research and is paving the way to creative healthcare solutions. Its available surface area, size, and sensitive surface provide it with high precision of medicines, improved drug delivery, and better targeting of medication.^1^ Green nanoparticle (NP) synthesis is now drawing considerable attention because of its low production costs, ease of manufacture, safety, and ecological compatibility. It is a trustworthy method for synthesizing a wide range of plants, bacterial, and fungal extract nanostructures, and even mixed salts such as metal salts. Green NP synthesis is a pragmatic and sustainable alternative to conventional synthesis processes.^2^ Plant extract-based green synthesis of nanoparticles is a sustainable, eco-friendly method for the production of biocompatible nanoparticles with various biomedical applications, though challenges in reproducibility, scalability, and toxicity evaluation remain.^3,4^ The green approach is replacing traditional chemical and physical methods of nanoparticle synthesis, which emit dangerous toxic chemicals.^5,6^ Green synthesized nanoparticles are utilized in the cosmetics and food industries as better, safer, and more environmentally friendly compounds. They have uses in agricultural, photocatalytic, sensor, biomedical, and tracking of various chemicals.^7,8^

The most popular category of metallic nanoparticles is the zinc oxide nanoparticles (ZnONPs) because they are characterized by a high surface-to-volume ratio, low toxicity, high chemical stability, and low costs. The ratio may be used in numerous biomedical disciplines, such as antiglycation,^9^ tissue engineering, medication delivery, biosensing, wastewater remediation, and agricultural enhancement.^10,11^ It acts as a potent photocatalyst in the dye degradation under sunlight. Furthermore, Ag-doped nanomaterials exhibit a wide spectrum of unique properties in a variety of biological applications.^12,13^ Bimetallic nanoparticles (BNPs) offer enhanced properties compared to monometallic nanoparticles, such as improved electronic, catalytic, and biological characteristics.^14^ The metal–metal NCs have attracted more attention in the research field because a Schottky barrier is achieved when the metal oxides are bound with metals, where the electrons in the oxide charge to the metal until equilibrium is achieved, leading to bending of the conduction band. Formation of a Schottky barrier reduces the photoexcited electron–hole recombination. The localized surface plasmon resonance of metals also contributes to the enlargement of the light absorption range of the metal oxides and consequently enhances their performance when used in photocatalytic and solar processes.^15^ Green synthesis of BNPs, particularly through plant-mediated methods, is drawing attention due to its avoidance of harsh conditions, an eco-friendly approach, and harmful chemicals, while producing biocompatible nanoparticles capped by therapeutically important plant metabolites.^16^

Biofabricated Ag@ZnO NCs exhibit strong antibacterial potential against a range of Gram-negative and Gram-positive strains of bacteria due to enhanced ROS generation and membrane disruption. They also show anti-inflammatory effects by activating inflammatory markers, antioxidant potential through free-radical scavenging, and antiglycation potential, which is useful in managing carbohydrate-related metabolic effects. Additionally, Ag@ZnO NCs demonstrate cytotoxic effects against cancer cells^17^ and anti-hemolytic properties, indicating reduced damage to red blood cells under stress conditions.^18^

*Taxus wallichiana Zucc*. (Himalayan yew) a member of the family Taxaceae, it grows in the Indian Himalayan forest and is a slow-growing understory plant that grows in altitudes between 1800 and 3300 m above mean sea level. Several research studies have been done on the medicinal nature of the Himalayan yew. *T. wallichiana* has elicited much interest in the scientific community because its leaves and bark have been discovered to contain high levels of “taxol.” Taxol is well known for its anti-cancer effects^19,20^ and is efficacious in treating breast and ovarian cancers.^21,22^*T. wallichiana* also contains a polyoxygenated cyclic diterpenoid called paclitaxel, which has gained significant attention due to its anticancer potential against breast, pancreatic, lung, ovarian, and cervical cancer.^23,24^ In ancient times, native peoples used this plant widely for traditional treatments to cure many disorders such as headache, fever, diarrhea, nervous system disorders, and fractures, *etc., T*. *wallichiana* exhibits quite high antioxidants and high anti-microbial activity, as well as secondary metabolites.^25,26^ The bark and leaves of this plant are extensively utilized by indigenous people to make tea and for various traditional medicinal purposes. *T*. *wallichiana* is a rich source of pharmacologically active chemicals like paclitaxel. This plant has yielded about 170 chemicals, including diterpenoids, taxanes, lignans, phenolics, and flavonoids.^27,28^

This research aligns with the global shift toward sustainable chemistry and the United Nations Sustainable Development Goals (SDGs), specifically SDG 3 (Good Health and Well-being) and SDG 12 (Responsible Consumption and Production). Despite the growing interest in nano-biotechnology, studies on *T*. *wallichiana*-capped bimetallic nanoparticles and their mechanistic comparison with monometallic counterparts remain scarce. The primary aim of this work is to establish a robust green synthesis protocol for silver (AgNPs) and zinc oxide (ZnONPs) nanoparticles, alongside their bimetallic Ag@ZnO nanocomposite (NC) integration, utilizing the leaf extract of *T*. *wallichiana*. The foundational blueprint of this project is strictly anchored in the paradigms of green chemistry, sustainable synthesis, and eco-friendly methodologies. This approach establishes a totally non-toxic, biocompatible biofabrication pathway that entirely bypasses the hazardous reducing agents, toxic surfactants, or harsh organic solvents typical of conventional chemical synthesis. As an ethnopharmacologically significant species, *T. wallichiana* possesses a rich phytochemical repertoire that facilitates the efficient reduction, stabilization, and capping of these nanomaterials. Specifically, active secondary metabolites such as flavonoids, phenolics, and terpenoids serve a dual role in the reaction matrix. Their functional groups (such as hydroxyl and carbonyl groups) act as reducing agents by readily donating electrons to reduce the precursor metal ions (Ag^+^ and Zn^2+^) into their stable atomic forms (Ag^0^ and ZnO). Following nucleation, these biomolecules behave as capping and stabilizing ligands, structurally coordinating around the primary particle cores *via* steric hindrance and electrostatic interactions to regulate particle growth and minimize uncontrolled macro-aggregation. This study investigates the role of these bioactive compounds in dictating the physicochemical properties and colloidal stability of the resulting particles. Critically, this research represents the first systematic study to synthesize and comparatively evaluate Ag@ZnO bimetallic systems against monometallic ZnO and Ag nanoparticles using *T. wallichiana*. By demonstrating the synergistic effects of the bimetallic architecture, this work advances the potential application of plant-mediated nanomaterials in phytomedicine, environmental remediation, and biomedical engineering, offering a sustainable alternative to traditional chemical synthesis.

## 2 Materials and methods

### 2.1 Plant collection

**Fig. 1:**
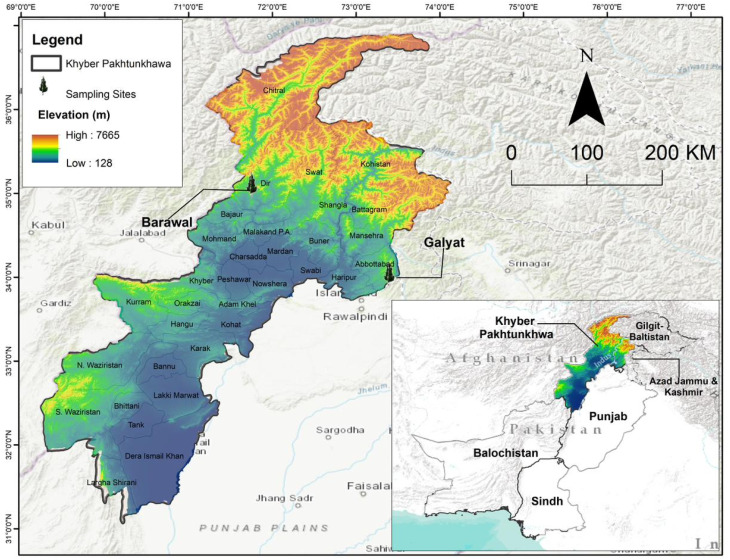
Map showing the location of *T. wallichiana* sampling sites across the Barawal (Dir Upper) and Galyat (Abbottabad) regions of Khyber Pakhtunkhwa, Pakistan.

*T*. *wallichiana* plant bark, branches, and leaves were collected in Northern Pakistan (Barawal region of Upper Dir and Galyat region) in April 2025, and this collection was performed on private land, with explicit permission from the landowner. All activities adhered to institutional, national, and international ethical guidelines for the collection of plant materials. The taxonomic identification was carried out at Molecular Systematic and Applied Ethnobotany Laboratory, Quaid-i-Azam University by Prof. Zabta Khan Shinwari, an expert taxonomist. A voucher specimen (no. 134402) was deposited in the Herbarium of Pakistan (ISL) for future reference. Plant samples were also stored in clean, ventilated bags to prevent cross-contamination and the buildup of humidity before being taken to the laboratory. The plant collection was carried out in April, when the weather is ideal for peak growth, resulting in the highest levels of bioactive compounds. Bark, branches, and leaves of *T*. *wallichiana* were harvested, but only the leaves were used for the preparation of extracts and synthesis of nanoparticles because they are reported to contain higher levels of bioactive phytochemicals, including flavonoids, phenolics, and paclitaxel. The geographic locations and coordinates of the *T. wallichiana* leaf sampling sites across the Barawal (Dir Upper) and Galyat (Abbottabad) regions of Khyber Pakhtunkhwa, Pakistan, are illustrated in Fig. 1.

### 2.2 Plant extraction

Samples were then washed in the laboratory with distilled water to remove dust, grime, and other foreign contaminants. Thus, only the leaf powder was used for aqueous extract in the biosynthesis of nanoparticles, as it has a higher phytochemical content than the bark and branches. Afterwards, the leaf aerial sections were shade-dried at room temperature to avoid damaging the phytochemical ingredients. Upon drying, the dried aerial parts were subjected to a commercial grinder for conversion to fine powder.^29,30^ About 20 gm of finely ground leaf powder was kept in a beaker containing 200 mL of double-distilled water. The mixture was heated at 60 °C under continuous stirring for 3 hours.^31^ While organic solvents like ethanol are known to be highly efficient for extracting hydrophobic phenolic compounds, hot-water extraction was intentionally selected to align strictly with the principles of green chemistry and to mirror the traditional ethnomedicinal preparations of *T. wallichiana*. This approach ensures an entirely eco-friendly, non-toxic extraction pathway free of hazardous organic solvent residues. The extract was cooled down. The resulting crude extract was then filtered *via* Whatman no. 1 filter paper and muslin cloth.^32^

### 2.3 Biosynthesis of AgNPs, ZnONPs, and Ag@ZnO nanocomposites

A volume of 200 mL of *T. wallichiana* leaf extract was combined with 100 mL of 0.01 M AgNO_3_ solution.^33^ The sample was stirred at 1000 rpm at 60 and centrifuged for 15 min at 12 000 rpm.^34^ The pH of the solution was raised from 6.7 to 11 by adding 0.2 M NaOH; the color altered from colorless to dark brown, indicating the synthesis of nanoparticles.^35,36^ The hydroxide ions in solution promotes reduction and the formation of oxides, which results in the appearance of precipitates. 200 mL of *T. wallichiana* leaf extract was combined with 100 mL of 0.1 M zinc acetate solution and stirred at 800 rpm for 30 minutes at ambient temperature.^37^ The pH of the mixture was maintained at 12 by the addition of NaOH. Afterwards, the solution was swirled around at 65 °C, with the color turning slowly to a yellowish creamy white.^38^ 200 mL of *T. wallichiana* extract was added to 50 mL of 0.1 M zinc acetate solution, and 50 mL of 0.01 M silver nitrate was added dropwise 1 : 10 (Ag/Zn = 0.1).^17^ Then, 2 M sodium hydroxide was added gradually to bring the pH to 9 and stirred. After 4 hours, a dark brown precipitate formed. All precipitate was washed, centrifuged, dried at 60 °C, crushed, and calcinated at 450 °C for 3 hours.^39^ The solutions were centrifuged and treated with DI several times after the heat treatment. For later use, the well-dried sample was stored at 4 °C in the dark.
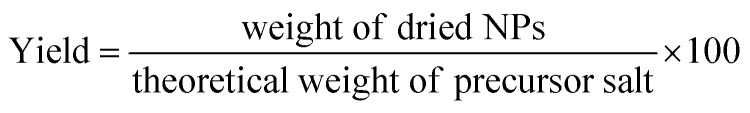


### 2.4 Characterization techniques

The surface plasmon resonance (SPR) peak of *T. wallichiana* base AgNPS, ZnONPs and Ag@ZnO was assessed using UV-visible spectroscopy (Model: UV-2600, Shimadzu, Japan) over the wavelength range 200–600 nm. An FTIR (Fourier transform infrared spectrometer; Model: Spectrum Two, PerkinElmer, USA) was used to identify chemical bonds and functional groups for reduction and stabilization; it was combined with KBr, pressed into pellets, and scanned in the 4000–500 cm^−1^ range. Crystalline nature and phase expansion of Ag@ZnO were analyzed using a Bruker D2-phaser advanced diffractometer using Cu-Kα radiation (*λ* = 1.5406 Å) by X-ray diffraction operating at 40 kV and 30 mA. The results of the photon correlation spectroscopy were determined at a scanning rate of 2 min^−1^ to measure the zeta potential in a 2*θ* range spanning 10 to 80. The mean zeta potential was observed within the 60 second analysis. Direct analysis of the dispersion without dilution was done. SEM and TEM were used to determine the morphology and size of the synthesized nanoparticles. In the quantitative elemental analysis of the data, EDX software was utilized. Dynamic light scattering (DLS) was used to measure the hydrodynamic diameter, as well as the zeta potential (Model: Zetasizer Nano ZS90, Malvern Instruments, UK).

### 2.5 Phytochemical analysis, antioxidants, cytotoxicity, and enzymatic inhibition

#### 2.5.1 Total flavonoid content (TFC)

The aluminium chloride colorimetric method was used to estimate the total flavonoid content. The NPs sample (1 mL) was put into 3 mL of 5% NaNO_3_, stirred with it, and left to stand for 1 min. An aliquot of 100 µl of 10% AlCl_3_ was put in it and incubated at room temperature, 5 minutes after which 500 mL of 1 M NaOH was added. The absorbance was recorded as 415 nm. Distilled water was taken as a blank. The outcomes are measured in µg of Quercetin equivalents per 100 g.^40^

#### 2.5.2 Total phenolic content (TPC)

The FC (Folin–Ciocalteu) colorimetric method was used to assess total phenolic content. 0.1 mL of FC reagent and 5 mL of the sample were agitated for 5 min. After the addition of 0.2 mL of 20% Na_2_CO_3,_ the samples were weighed thrice and incubated in the dark at room temperature for 60 minutes. The absorbance was recorded at 765 nm. A blank was made with distilled water. The findings are calculated in µg of gallic acid equivalents per 100 g.^40^

#### 2.5.3 The antioxidant activities

The antioxidant activities were quantified by using radical scavenging, *i.e.*, DPPH, ABTS, and reducing powers, *i.e.*, FRAP, and TAC assays by following established protocols. Serial dilutions of two-fold were used (62.5–1000 µg mL^−1^) for each sample from their stock solution, *i.e.*, 1000 µg mL^−1^.

#### 2.5.4 DPPH radical scavenging assay

The free RS action of the *T. wallichiana*-mediated AgNPs, ZnONPs, and Ag@ZnO NCs was determined. 100 µL of different concentrations of monometallic and bimetallic nanoparticles (62.5–1000 µg mL^−1^). As the free radical source, 3 mL of a DPPH (2,2-diphenyl-1-picrylhydrazyl) solution (0.1 mM in methanol) was added and left to stand at room temperature for 30 minutes. The absorbance was taken at 517 nm using a UV-vis spectrophotometer. Ascorbic acid was employed as a synthetic antioxidant. The DPPH activity was determined using the following formula.^41^
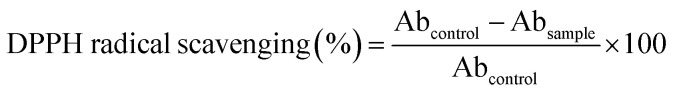
where Ab_control_ is the absorbance of the DPPH reagent, while Ab_sample_ is the DPPH solution with the sample absorbance.

#### 2.5.5 ABTS˙^+^ radical scavenging activity

The ABTS˙^+^ (2,2′-azino-bis(3-ethylbenzothiazoline-6-sulfonic acid)) RS assay was adapted with some modifications.^42^ Briefly, the working solution of ABTS˙^+^ radical was prepared by adding 2.45 mM potassium persulfate to a 7 mM ABTS˙^+^ solution at a 1 : 1 ratio, and this was left at RT in the dark for 16 h. Ethanol was added to the working solution to dilute it, and the absorbance at 734 nm was recorded using a microplate reader. The sample (20 mL) and ABTS˙^+^ working solution (180 mL) were pipetted into each well. The mixture was left to incubate in the dark for 6 min at room temperature, after which the absorbance was read at 734 nm. The calculation of ABTS˙^+^ radical scavenging was based on.
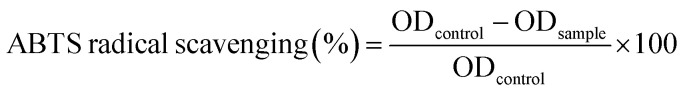


In which OD _control_ is the absorbance of the ABTS˙^+^, and OD _sample_ is the absorbance of ABTS˙^+^ with the sample.

#### 2.5.6 Ferric reducing antioxidant power (FRAP)

The antioxidant power of Tw-mediated Ag, ZnO, and Ag@ZnO NCs was evaluated using the FRAP (Ferric Reducing Antioxidant Power) method,^43^ with slight modifications. NPs were synthesized at various concentrations of (62.5–1000 µg mL^−1^). For each assay, 1 mL of sample was added to 1 mL of potassium ferricyanide and 1 mL of PBS (0.2 M, pH 6.6). The sample was incubated at 20 min 50 °C, after which 0.5 mL of TCA (10% w/v) was added to the reaction to stop it. The mixture was centrifuged for 10 min at 3000 rpm, and the resultant supernatant was mixed with 0.125 mL of ferric chloride and 0.5 mL of deionized water. Absorbance was taken at 700 nm using a UV-vis spectrophotometer. Ascorbic acid was taken as a standard. Results are reported in the form of mean ± standard deviation.

#### 2.5.7 Total antioxidant capacity (TAC)

The TAC of the *T*. *wallichiana* mediated AgNPs, ZnONPs, and Ag@ZnO NCs was measured as described by Azzi *et al.*^43^ Gallic acid was taken as the positive control. For each sample, 100 µL of NPs solution at serial dilutions (62.5–1000 µg mL^−1^) was added to 1 mL of reaction mixture consisting of 28 mM sodium phosphate, 0.6 M sulfuric acid, and 4 mM ammonium molybdate. The sample mixtures were incubated at 95 °C in a boiling water bath for 1 h and 30 minutes. After incubation, the sample was cooled at RT, and the absorbance was recorded at 695 nm against a blank. Results are reported as mean ± standard deviation.^44^

#### 2.5.8 Antibacterial activity

The antibacterial activity of *T. wallichiana*-mediated AgNPs, ZnONPs, and Ag@ZnO NCs was evaluated following the protocol using the disc diffusion method. Four MDR bacterial strains were selected, including two Gram-positive, *i.e.*, *Streptococcus pneumoniae* (ATCC#6538) and *Bacillus subtilis* (ATCC#6633), and two Gram-negative, *i.e.*, *Klebsiella pneumoniae* (ATCC# 4619) and *Escherichia coli* (ATCC 15,224). Strains were cultured on Muller–Hinton agar and incubated at 37 °C for 24 h.100 µL of each strain was swabbed onto agar plates (100 µg mL^−1^).^45^ Plates were incubated for 24 h at 37 °C. A vernier calliper was used to measure the zones of inhibition to evaluate antibacterial activity.

#### 2.5.9 Anti-inflammatory activity

A 3 mL reaction mixture in 2 mL of PBS buffer (phosphate-buffered saline), consisting of a pH of 6.4, 0.5 mL of 1% bovine albumin, and 0.5 mL of NPs at concentrations of 62.5–1000 µg mL^−1^ was added to the reaction mixture. The sample was incubated for 15 min in a water bath at 37 °C, then heated at 70 °C for 5 min. Turbidity at 660 nm was measured using a microplate reader after the cooling process. PBS was taken as the control, and 1000 µg of diclofenac sodium was taken as the standard.^46^ The percentage inhibition of protein denaturation was measured using the following formula:
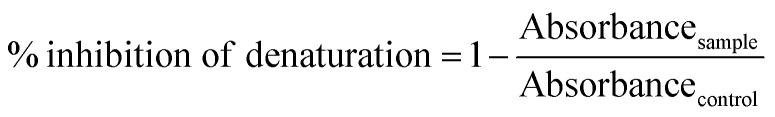


#### 2.5.10 α-Amylase inhibition activity

Anti-diabetic activity of *T. wallichiana*-based AgNPs, ZnONPs, and Ag@ZnO NCs was measured in terms of α-amylase inhibition. The α-amylase assay was performed according to the method described by ref. 18, using porcine pancreatic α-amylase (Type VI-B, ≥5 units per mg solid, Product no. A3176, Sigma-Aldrich, St. Louis, MO, USA). Briefly, 15 µg mL^−1^ of the plant extract/nanoparticles at different concentrations (50–200 µg mL^−1^) diluted in phosphate buffer was added to 5 µL of the porcine pancreatic α-amylase enzyme solution in a 96-well plate. Following a 10 min incubation period at 37 °C, the reaction was initiated by adding 20 µL of starch solution and incubated further for 30 min at 37 °C. The reaction was subsequently terminated by the addition of 10 µL of 1 M HCl to each well, followed by 75 µL of iodine reagent. A blank containing phosphate buffer (pH 6.9) instead of the extract, alongside a positive control (acarbose, 64 µg mL^−1^), was prepared. No-enzyme and no-starch controls were included for each test sample. The absorbance was recorded at 580 nm, and the percentage inhibitory activity was calculated using the following equation:
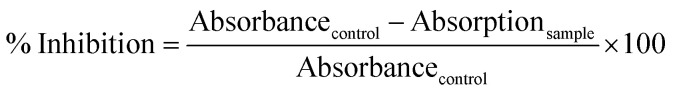


#### 2.5.11 Biocompatibility with human red blood cells (hRBCs)

Hemocompatibility of *T*. *wallichiana*-mediated Ag, ZnO, and Ag@ZnO NCs was measured with RBCs of blood samples from healthy males and females (ages 28–35 years) after taking their written consent. EDTA vacutainers were used to collect blood samples to prevent clotting. After extracting RBCs, 100 mL NPs and erythrocytes were taken in an Eppendorf Tube, then incubated at 37 °C for 1 h and proceed to centrifuged at 10 000 rpm for 10 minutes. A 96-well plate was filled with 100 mL supernatant. The release of haemoglobin at 540 nm was measured by using a BioTek ELX800 Absorbance Microplate Reader (BioTek Instruments, France). Triton X-100 and DMSO acted as positive and negative controls, respectively. The results were expressed as % haemolysis, which was calculated by the following formula:
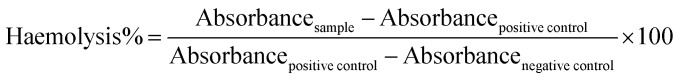


#### 2.5.12 Lethality against brine shrimp

Tw-mediated AgNPs, ZnONPs, and Ag@ZnO NCs toxicity to *Artemia salina* (brine shrimp) was done in a 96-well plate over 24 h. To begin with, the egg of *A. salina* was hatched by incubating the eggs in a 24–48 h plastic tray with 38 g L^−1^ sterile seawater topped with dried yeast (6 mg L^−1^) and an adequate supply of oxygen. Ten full-grown nauplii (phototropic) were collected, and NPs (100–400 µg mL^−1^) were added to the well. The final volume was brought to 100 mL. Doxorubicin and 1% DMSO in seawater were used as positive control and negative control, respectively. LC_50_ (Lethality Concentration) was calculated by using NPs with 50% mortality.
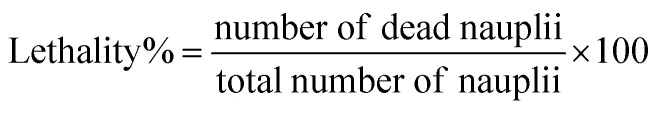


#### 2.5.13 Phytotoxicity

To study the phytotoxicity of *T. wallichiana*-mediated AgNPs, ZnONPs, and Ag@ZnO NCs, the morphological parameters of radish seeds were evaluated under the application of Ag, ZnONPs, and Ag@ZnO NCs. Healthy seeds received from the NARC (National Agriculture Research Centre) in Islamabad, Pakistan, were subjected to surface sterilization for 5 minutes with 3% sodium hypochlorite before being washed thrice with distilled water. The seeds were then sown in Petri dishes under ideal conditions (65% humidity, a 5LS light period, RH 60%, and +25 °C temperature). AgNps, ZnONPs, and Ag@ZnO NCs at a concentration of 100, 200 300 and 400 µg mL^−1^ were used as a treatment of three to four-day-old wheat seedlings (two-leaf stage). The effects on morphology (seed germination and root/shoot length) were recorded after treatment for three consecutive days. The experiment was performed in triplicate.^47^

### 2.6 Statistical analysis

Statistical analyses were executed using GraphPad Prism 9.0.0, where a two-way ANOVA test followed by Tukey's multiple comparison analysis was applied to evaluate significant differences among treatments. All the experimental results were first keyed and tabulated in Microsoft Excel. Principal Component Analysis (PCA) was used as a multivariate assessment in RStudio, employing the ggbiplot, ggplot2, and corrplot packages. The other post hoc comparisons were performed using the Tukey HSD test (*p* < 0.05; 95% confidence level) in GraphPad Prism. To have a high-quality presentation of the results, the graphs, as well as instrumental characterization results, such as XRD, UV-vis, and FTIR spectra, were perfected with the help of OriginPro 2026.

## 3 Results

### 3.1 Physicochemical characterization of synthesized nanoparticles

#### 3.1.1 Ultraviolet-visible spectroscopy (UV-vis)

**Fig. 2:**
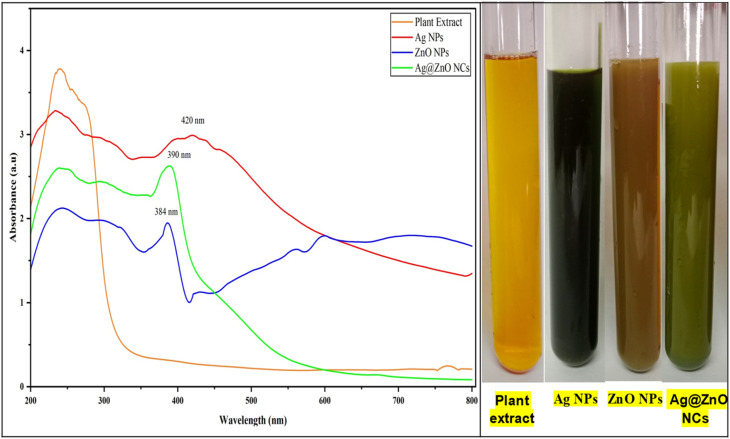
UV-vis spectra of green-synthesized AgNPs, ZnONPs, and Ag@ZnO NCs.

The UV-vis absorption spectra of the plant extract exhibit a high absorption peak at 230–250 nm, which means that the plant extract contains aromatic compounds and bioactive phytochemicals. The absorption peak of ZnO-NPs is at 379 nm, and the surface plasmon resonance (SPR) is at 422 nm in AgNPs (Fig. 2). The SPR peak supports the fact that Ag^+^ ions are reduced to metallic Ag nanoparticles. The Ag@ZnO NCs exhibit a wider range of absorption spectra with a maximum of 390 nm, which is slightly red shifted from that of single AgNPs. This transformation reveals that the nanocomposite was formed correctly. The observed spectrum characteristics confirm the successful fabrication of ZnO, Ag, and Ag@ZnO nanoparticles using the plant extract as a green reducing and capping agent. These UV-vis measurements can be considered as the primary evidence of the structural and functional analysis of the produced nanoparticles.

#### 3.1.2 Fourier transform infrared spectroscopy (FTIR)

**Fig. 3:**
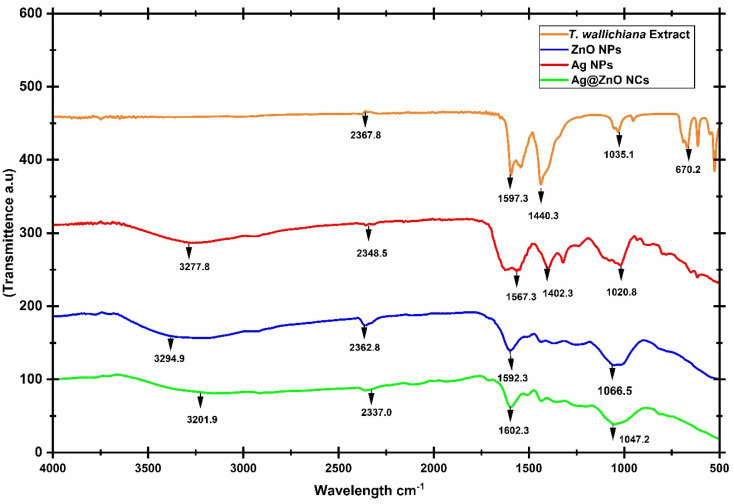
FTIR spectra of the pristine *T. wallichiana* leaf extract compared with the biofabricated monometallic AgNPs, ZnONPs, and bimetallic Ag@ZnO NCs.

The FTIR spectra of *T. wallichiana* extract, AgNPs, ZnONPs, and Ag@ZnO bimetallic nanoparticles were obtained at 400–4000 cm^−1^ range to identify the functional groups that assist in the formation and stability of the nanoparticles (Fig. 3). The presence of plant-based biomolecules as reducing and capping agents is evident in a broad absorption band at about 3200–3300 cm^−1^ across all spectra, corresponding to the O–H stretch of phenolic and alcoholic groups. The bands between 2337–2367 cm^−1^ can be linked to either the triple bond stretching or CO_2_. The bands of high strength at 1567–1602 cm^−1^ are related either to C=O stretching (amide I) or aromatic C=C vibrations, which presuppose a component of proteins and polyphenolic chemicals in the stability of nanoparticles. The peaks at 1402–1440 cm^−1^ are assigned to C–H bending and symmetric stretching vibrations of carboxylate groups, indicating the presence of organic components attached to the nanoparticle surface. Also, peaks at 1020–1066 cm^−1^ are attributed to C–O stretching vibration of alcohols, ethers, or polysaccharides. The minimal differences in the peak positions in the Ag@ZnO spectrum relative to those of individual Ag and ZnO nanoparticles indicate the presence of biomolecular interactions with the metal surface and demonstrate the effective formation and stabilization of the bimetallic nanostructure.

#### 3.1.3 Transmission electron microscopy (TEM), SAED and particle size distribution analysis

**Fig. 4:**
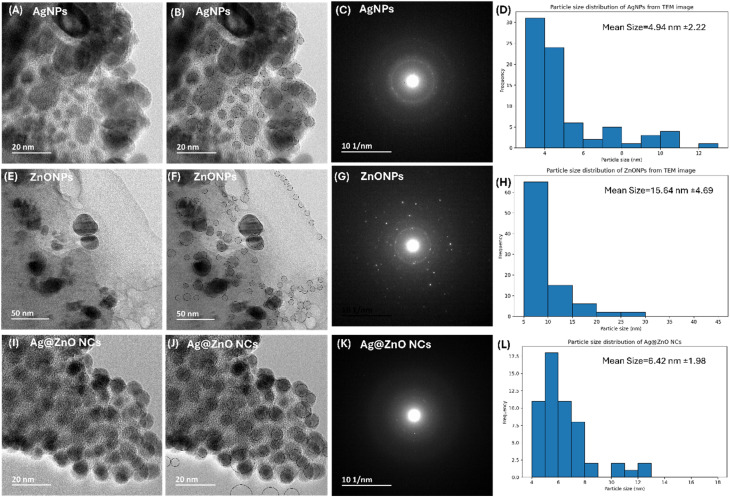
TEM, SAED, and particle size distribution analysis of biosynthesized nanoparticles. (A–D) AgNPs, (E–H) ZnONPs and (I–L) Ag@ZnO NCs.

TEM, selected area electron diffraction (SAED), and particle size distribution analyses were performed to investigate the morphology, crystallinity, and size characteristics of the biosynthesized AgNPs, ZnONPs, and Ag@ZnO nanocomposites (Fig. 4). The TEM micrographs revealed that all samples consisted of nanoscale particle domains exhibiting varying degrees of aggregation, which is commonly observed in phytochemically synthesized nanomaterials due to strong interparticle interactions and the presence of surface-bound biomolecules. The AgNPs (Fig. 4A–D) appeared as densely packed aggregates composed of small electron-dense nanodomains with predominantly spherical to quasi-spherical morphology. Although individual particle boundaries were partially obscured by overlapping and clustering, ImageJ-assisted analysis (Fig. 4B) enabled estimation of the particle size distribution. The histogram (Fig. 4D) showed that most particles were concentrated within the 3–8 nm range, with an average particle diameter of 4.94 ± 2.22 nm. The corresponding SAED pattern (Fig. 4C) exhibited concentric diffraction rings with bright diffraction spots, confirming the crystalline and polycrystalline nature of the Ag nanoparticles. ZnONPs (Fig. 4E–H) exhibited larger particle domains with spherical, quasi-spherical, and slightly oval morphologies. The nanoparticles were distributed throughout a thin amorphous matrix and displayed localized aggregation. The annotated TEM image (Fig. 4F) highlighted the measurable particle boundaries used for size estimation. Particle size distribution analysis (Fig. 4H) revealed a broader size range than that observed for AgNPs, with an average particle diameter of 15.64 ± 4.69 nm. The SAED pattern (Fig. 4G) displayed distinct concentric diffraction rings characteristic of a well-crystallized polycrystalline ZnO phase, indicating successful formation of crystalline ZnONPs. The Ag@ZnO nanocomposites (Fig. 4I–L) showed closely packed nanosized domains with improved uniformity relative to pure ZnONPs. The particles appeared predominantly spherical and were arranged as compact aggregates. The ImageJ-assisted analysis (Fig. 4J) confirmed the presence of relatively homogeneous particle domains, while the histogram (Fig. 4L) demonstrated a narrower particle size distribution with an average diameter of 6.42 ± 1.98 nm. The SAED pattern (Fig. 4K) showed bright concentric diffraction rings and diffraction spots, confirming the crystalline polycrystalline nature of the nanocomposites and supporting the successful integration of Ag and ZnO phases within the composite structure. Overall, TEM analysis confirmed the formation of nanoscale AgNPs, ZnONPs, and Ag@ZnO NCs with distinct particle size distributions and varying aggregation behavior. The average particle size followed the order ZnONPs (15.64 ± 4.69 nm) > Ag@ZnO NCs (6.42 ± 1.98 nm) > AgNPs (4.94 ± 2.22 nm). Furthermore, the SAED patterns of all samples demonstrated characteristic diffraction rings, confirming their crystalline and polycrystalline structures. These findings are consistent with the successful biosynthesis of crystalline nanomaterials and the formation of Ag-decorated ZnO nanocomposites.

#### 3.1.4 Scanning electron microscope (SEM)

**Fig. 5:**
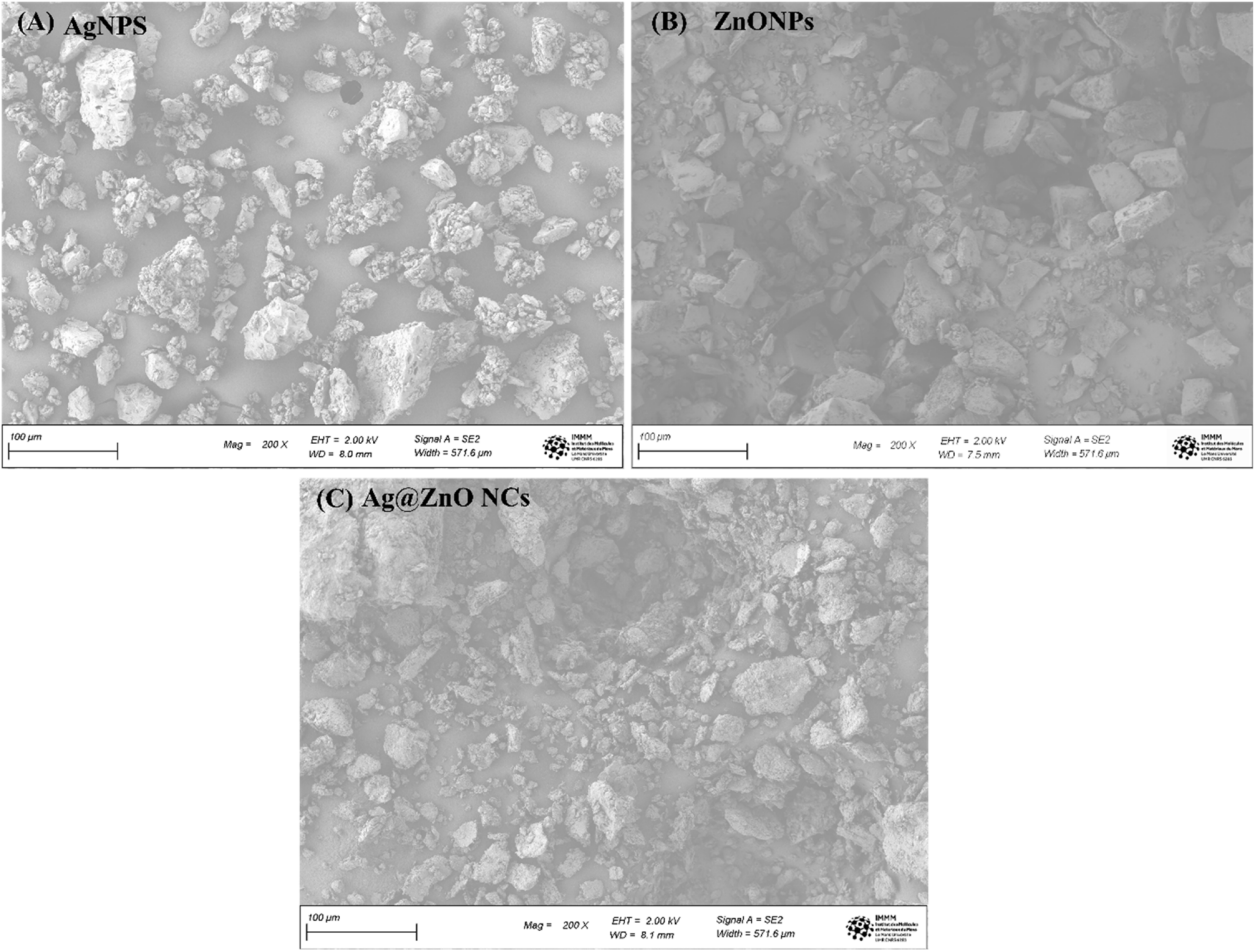
SEM micrographs showing the surface morphology of biofabricated nanoparticles: (A) AgNPs with irregular granular and rock-like aggregated morphology, (B) ZnONPs with dense blocky, flake-like, and plate-like aggregates, and (C) Ag@ZnO nanocomposites showing heterogeneous rough aggregates with mixed granular structures. Scale bar = 100 µm; magnification = 200×.

The macrostructural features, surface texture, and wide-field distribution of the biofabricated powder samples were examined using SEM, as shown in Fig. 5. At low magnification (200×) with a standardized 100 µm scale bar, all three systems, including AgNPs (Fig. 5A), ZnONPs (Fig. 5B), and Ag@ZnO NCs (Fig. 5C), exhibited an irregular, polydisperse, and highly aggregated particulate morphology. The AgNPs displayed rough, rock-like granular aggregates with non-uniform particle distribution, whereas the ZnONPs showed comparatively dense, blocky, flake-like, and plate-like agglomerated structures. In contrast, the Ag@ZnO NCs revealed a heterogeneous mixed morphology, consisting of irregular granular particles distributed over larger ZnO-like aggregates, suggesting the successful integration of Ag with the ZnO matrix. The observed aggregation may be attributed to the high surface energy of the nanoparticles, phytochemical capping by *T. wallichiana* secondary metabolites, and particle–particle interactions during drying and powder preparation. The rough and uneven surface morphology of the Ag@ZnO NCs indicates the development of a more complex surface architecture compared with the individual AgNPs and ZnONPs. Such surface heterogeneity may provide additional active sites, which could be beneficial for catalytic, antimicrobial, or other functional applications. Overall, SEM analysis confirmed that the synthesized nanoparticles were not present as isolated individual particles but rather as aggregated micro-scale clusters composed of smaller primary nanostructures.

#### 3.1.5 Energy dispersive X-ray (EDX)

**Fig. 6:**
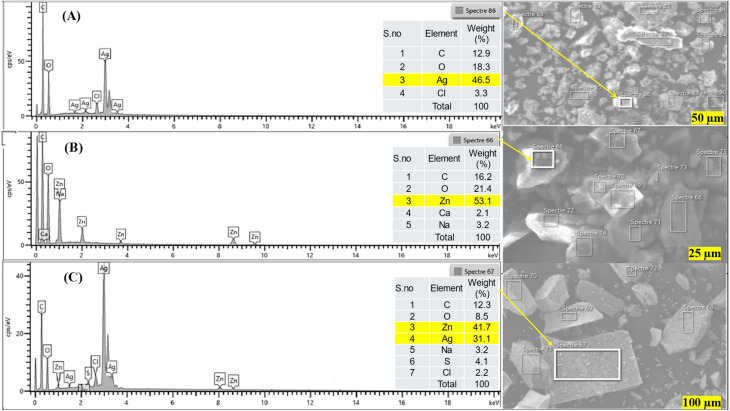
SEM-EDS analysis of AgNPs (A), ZnONPs (B), and Ag@ZnO NCs (C), confirming the elemental mapping and incorporation of Ag and Zn within the synthesized nanostructures.

The EDX spectra acquired from 0–20 keV confirms the elemental signatures of the produced nanomaterials. In the AgNPs sample, powerful Ag peaks arise between 2.5–3.5 keV, corresponding to Ag Lα and Lβ emissions, contributing 46.5 wt%. Minor C, O, and Cl peaks indicate organic remains of plants and trace pollutants (Fig. 6A). The strong peaks of ZnO are 1.0–1.2 keV and 8.5–9.5 keV, and the Zn content is 53.1 wt%. O, Ca, and Na are also impurities in the extract (Fig. 6B). Zn (1.0–9.0 keV) and Ag (2.5–3.5 keV) peaks indicate that Ag@ZnO nanocomposites are successfully prepared with a Zn proportion of 41.7% and 31.1%, respectively, Fig. 6C. The irregular Ag particles, compact ZnO crystallites, and irregular composite structures in Ag@ZnO are observed in the SEM pictures. Altogether, the EDX and SEM analysis confirmed the anticipated elemental patterns, the bimetallic formations, and trace impurities of biogenic synthesis. The elemental signatures and purity profiles of the biofabricated nanomaterials were verified *via* EDX spectroscopy across an acquisition range of 0–20 keV, as shown in Fig. 6. In the monometallic AgNPs spectrum (Fig. 6A), powerful, characteristic silver peaks emerge distinctly between 2.5 and 3.5 keV, corresponding precisely to the silver Lα and Lβ shell emissions and confirming an elemental silver contribution of 46.5 wt%. Minor accompanying peaks for carbon (C), oxygen (O), and chlorine (Cl) represent the organic phytochemical residues originating from the *T*. *wallichiana* extract shell along with trace processing components. For the ZnONPs sample (Fig. 6B), the dominant electronic emissions are centered at 1.0–1.2 keV and 8.5–9.5 keV, verifying a substantial zinc (Zn) content of 53.1 wt%. Minor contributions from oxygen (O), calcium (Ca), and sodium (Na) are also present, reflecting benign biogenic elements co-extracted from the plant matrix. For the bimetallic Ag@ZnO nanocomposites (Fig. 6C), the simultaneous presence of both zinc lines (1.0–9.0 keV) and silver lines (2.5–3.5 keV) within the same spectrum proves the successful fabrication of the hybrid composite structure, revealing relative proportions of 41.7 wt% for Zn and 31.1 wt% for Ag, respectively. These elemental signatures correspond tightly with the surface morphologies captured in the accompanying SEM micrographs. The images track a clear structural transition from individual irregular silver particles and compact, distinct ZnO crystallites into fully integrated, irregular bimetallic heterostructures in the final Ag@ZnO composite. Taken together, the combined EDX and SEM data confirm the expected elemental patterns, successful bimetallic integration, and the characteristic biogenic surface-capping profile typical of plant-mediated synthesis.

#### 3.1.6 X-ray diffraction (XRD)

**Fig. 7:**
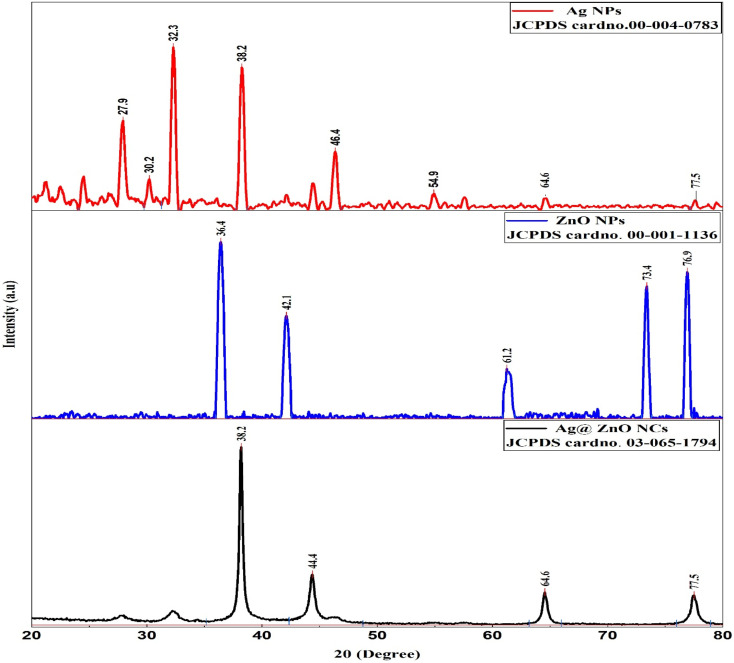
X-ray diffraction of green-synthesized AgNPs, ZnONPs, and Ag@ZnO NCs showing the diffraction peaks and confirming crystalline nature and matching the standard JCPDS cards.

The broadening of the XRD peaks indicates the nanocrystalline nature of the Ag@ZnO NCs. The most intense peak was used to determine the crystallite size of Ag@ZnO NCs using the Debye–Scherrer formula.
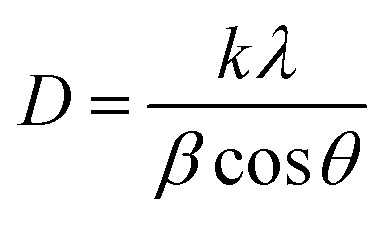
where *D* denotes the crystallite size (nm), *λ* represents the X-ray wavelength (Kα = 0.154 nm), *β* represents the full width at half maximum (FWHM) at the sharp intense peak measured in radians, and *θ* is the peak angle. Through this formula, the respective crystallite sizes for monometallic ZnO and AgNPs were 14.9 nm and 15.3 nm, respectively. The reaction mixture containing both salts resulted in a change of nanoparticle size to 17.6, depending upon the salt used (Fig. 7). It was observed that the addition of zinc salt at a higher concentration than silver salt increased the size of bimetallic NPs.

The XRD pattern of the AgNPs displayed four intense reflections at 2*θ* = 38.23, 44.38, 64.60, and 77.50, corresponding to the (111), (200), (220), and (311) planes of fcc silver, consistent with JCPDS 00-004-0783. The ZnONPs exhibited distinctive peaks at 35.7, 47.6, 58.9, and 69.6, which correspond to the (101), (102), (110), and (201) planes of hexagonal wurtzite ZnO, confirming strong crystallinity. The primary Ag peaks reappeared in the Ag@ZnO bimetallic nanocomposites at 38.19, 44.39, 64.55, and 77.50, with overlapping contributions from ZnO, confirming the coexistence of both crystal phases and a successful production of the composite structure (JCPDS 03 065 1794). The small broadening and intensity fluctuation in the composite pattern suggests nanoscale interactions between the Ag and ZnO domains.

#### 3.1.7 Zeta potential

Monometallic Ag nanoparticles had the biggest hydrodynamic size, *Z* average 422.8 nm, a broad bimodal distribution, and a high PDI 0.757, indicating strong aggregation in suspension. ZnO nanoparticles had a smaller average size of 377.2 nm and a lower PDI (0.416), indicating modest polydispersity but prominent aggregation. The bimetallic Ag@ZnO nanocomposites had the smallest mean size (*Z* average 195.6 nm) with a broad distribution (PDI 0.985) due to biogenic capping (Fig. S1A–C). Surface charge measurements revealed that AgNPs −18.3 mV and ZnONPs −16.8 mV had limited electrostatic stabilization, while Ag@ZnO had a greater negative potential −23.0 mV, indicating better colloidal stability (Fig. S1D–F). Overall, the bimetallic nanoparticles exhibited better charge-based stabilization and a smaller hydrodynamic size than their monometallic counterparts, while retaining the strong polydispersity associated with green synthesis. It is noted that the low surface charge of the synthesized primary particles causes weak dispersion stability, although the primary particle size is in the nanometer scale. This is why the suspension does not retain separate nanosized particles, even though the apparent contradiction with the formation of nanosized particles and their obvious agglomeration seems hard to explain.

### 3.2 Phytochemical analysis

#### 3.2.1 Total phenolic contents (TPC)

The total flavonoid content (TFC) was quantified with the help of a gallic acid calibration curve (0–120 µg mL^−1^), which showed good linearity with the *y* = 0.0047*x* + 0.7756 equation and an *R*^2^ value of 0.854 (Fig. S2B). The graph depicts the total phenolic content of the plant extract along with nanoparticles. In the given figure, the plant extract demonstrated the highest phenolic content, with a TPC of 28.4 mg Quercetin per g DW at the highest concentration of 1000 µg mL^−1^, exhibiting its significant antioxidant activity. In comparison, Ag@ZnO NCs had a TPC of 23.60 mg Quercetin per g DW, followed by ZnONPs with a TPC of 22.81 mg Quercetin per g DW, and AgNPs with the lowest at 21.95 mg Quercetin per g, while the Plant Extract has the highest phenolic content, making them excellent choices for antioxidant application (Fig. S2A).

#### 3.2.2 Total flavonoid contents (TFC)

To determine the TFC of plant extracts and nanoparticles, a calibration curve (Fig. S2D) was created using gallic acid calibration standards (0 to 120 µg mL^−1^). The calibration curve for TFC (*y* = 0.0011*x* + 0.3333) had a coefficient of determination (*R*^2^) of 0.9135, indicating strong linearity over the concentration range examined. The TFC of plant extract with various concentrations (62.5 to 1000 µg mL^−1^) was determined using the calibration curve equation and expressed as mg GAE per g dry mass of the extract.

The results (Fig. S2C) depict the total flavonoid content of *T. wallichiana* extract, AgNPs, ZnONPs, and Ag@ZnO NCs at various concentrations. At 1000 µg mL^−1^, plant extract had the highest flavonoid concentration (52 mg GAE per g DW), followed by Ag@ZnO NCs (42.5 mg GAE per g DW), ZnONPs (26 mg GAE per g DW), and AgNPs (28 mg GAE per g DW). While at the lowest concentration, 62.5 plant extract, Ag@ZnO NCs, ZnONPs, and AgNPs (25.5, 8, and 14 mg GAE/g DW, respectively). Flavonoids increase antioxidant potential by scavenging free radicals, chelating metal ions, and lowering oxidative stress. In conclusion, plant extract had the highest flavonoid content, Ag@ZnO NCs displayed the highest antioxidant activity, making them the most efficient of the evaluated materials.

### 3.3 *In vitro* biological assays of green-synthesized nanoparticles

#### 3.3.1 ABTS˙^+^ radical scavenging activity

The graph (Fig. S3A) of ABTS radical scavenging activities compares the antioxidant potential of plant extract, ascorbic acid, AgNPs, ZnONPs, and Ag@ZnO NCs at concentrations of 62.5 to 1000 µg mL^−1^. The strongest radical-scavenging activity was observed with ascorbic acid, which was close to 100, with an IC_50_ of 0.25 ± 4.8 µg mL^−1^ at higher doses. Plant extract and AgNPs showed 80% scavenging activity at 1000 µg mL^−1^ with an IC_50_ of 154 ± 5.3 µg mL^−1^. IC_50_ of 7.12 ± 4.10 µg mL^−1^, respectively. ZnONPs had a scavenging activity of 65% percent at the highest concentration with an IC_50_ of 196.5 ± 2.84 µg mL^−1^. Ag@ZnO NCs exhibited intermediate activity, reaching approximately 80% at 1000 µg mL^−1^, with an IC_50_ of 114.1 ± 3.42 µg mL^−1^. These data indicate that AgNPs have higher scavenging activity, with Ag@ZnO NCs performing better than ZnONPs alone.

#### 3.3.2 DPPH

The DPPH assay demonstrated a concentration-dependent increase in radical scavenging activity for all tested samples, with percentage inhibition increasing progressively from 62.5 to 1000 µg mL^−1^ DPPH. The Ag@ZnO NCs exhibited the highest percentage inhibition 80% at 1000 µg mL^−1^ (Fig. S3B), which was comparable to the standard ascorbic acid (75%), while plant extract, AgNPs, and ZnONPs showed comparatively lower inhibition at approximately 70%. Correspondingly, the Ag@ZnO NCs have the lowest IC_50_ value (206.5 ± 1.64 µg mL^−1^), significantly lower than ascorbic acid (357.3 ± 2.48 µg mL^−1^), plant extract (377.3 ± 4.46), AgNPs (659.1 ± 2.66 µg mL^−1^), and ZnONPs (762.4 ± 1.57 µg mL^−1^), indicating superior antioxidant efficiency. The enhanced percentage inhibition and lower IC_50_ of Ag@ZnO NCs confirm its stronger electron-donating ability and synergistic interaction between Ag and ZnO, which increases active surface sites for effective free radical neutralization.

#### 3.3.3 Ferric reducing antioxidant power (FRAP)

FRAP is a mechanism that is used to evaluate the capacity of antioxidants to reduce ions that show antioxidant activity. Although ascorbic acid was the most powerful against overall antioxidant strength, ZnONPs were the most efficient with the lowest IC_50,_ and Ag@ZnO NCs, and they can be considered as good candidates in antioxidant applications. At a concentration of 1000 µg mL^−1^, ascorbic acid had the highest ferric reducing antioxidant power of 90%, followed by Ag@ZnO NCs (88%), AgNPs (85%), ZnONPs (80%), and plant extract (75%). This reveals that whereas Ascorbic acid had the highest antioxidant activity, Ag@ZnO NCs surpass both AgNPs and ZnONPs. ZnONPs had the highest antioxidant efficiency, with the lowest IC_50_ value of 17.47 ± 2.905 µg mL^−1^, indicating the lowest concentration necessary to produce half-maximal reduction. Ag@ZnO NCs showed an intermediate IC_50_ value of 53.42 ± 6.21 µg mL^−1^ (Fig. S3C), while AgNPs had an IC_50_ of 71.78 ± 2.29 µg mL^−1^. Ascorbic acid exhibited the highest IC_50_ value (758.91 ± 4.70 µg mL^−1^), showing that it was less efficient than nanoparticles and plant extract with IC_50_ of 357 ± 2.48 µg mL^−1^.

#### 3.3.4 Total antioxidant capacity (TAC)

The Total Antioxidant Capacity (TAC) assay measures a substance's ability to destroy free radicals and protect against oxidative stress. At 1000 µg mL^−1^, ascorbic acid had the highest antioxidant activity (95%), followed by plant extract (75%), Ag@ZnO NCs (90%), AgNPs (85%), and ZnONPs (80%) (Fig. S3D). ZnONPs had the highest potency (IC_50_ = 0.052 ± 6.79 µg mL^−1^), requiring the lowest AgNPs, which had a lower IC_50_ value of 123.6 ± 7.22 µg mL^−1^, showing better efficiency than ascorbic acid, which had the highest IC_50_ value of 199.6 ± 6.278 µg mL^−1^. Ag@ZnO NCs, while exhibiting strong antioxidant activity (90%) at 1000 µg mL^−1^, had a higher IC_50_ of 702.0 ± 4.72 µg mL^−1^, making them less efficient than ZnONPs and AgNPs and plant extract, which is 357 ± 6.278 µg mL^−1^. In conclusion, while ascorbic acid showed the highest total antioxidant activity, ZnONPs exhibited the most efficient antioxidant properties, requiring the lowest concentration for maximum effect, making them the most potent among the tested substances in terms of dosage to produce half-maximal antioxidant activity.

### 3.4 Enzymatic inhibition

#### 3.4.1 α-Amylase inhibition assay

**Fig. 8:**
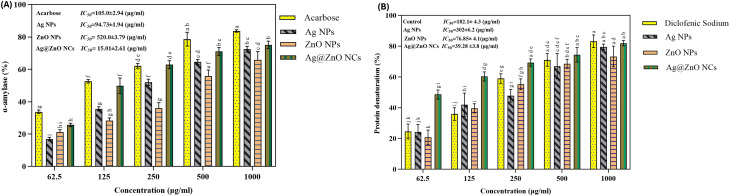
Bioactivity and evaluation of synthesized nanoparticles: (A) alpha-amylase inhibition, (B) protein denaturation.

The α-amylase inhibitory properties of acarbose (control), AgNPs, ZnONPs, and Ag@ZnO NCs became progressively more pronounced with concentration, with the highest inhibition noted at 1000 µg mL^−1^. At this maximum concentration (Fig. 8A), acarbose showed the highest percentage inhibition (83.9%), followed by Ag@ZnO NCs (76.8%), AgNPs (72.2%), and ZnONPs (65.8%). Although acarbose exhibited the greatest inhibition at the highest tested concentration, the IC_50_ values revealed a different potency trend. Ag@ZnO NCs demonstrated the lowest IC_50_ value (15.01 ± 2.61 µg mL^−1^), indicating the strongest inhibitory activity, followed by AgNPs (94.73 ± 1.94 µg mL^−1^) and acarbose (105.0 ± 2.94 µg mL^−1^), while ZnONPs showed the highest IC_50_ value (520.0 ± 3.79 µg mL^−1^), reflecting the weakest activity. Thus, although acarbose produced the highest percentage inhibition at 1000 µg mL^−1^, Ag@ZnO NCs exhibited superior inhibitory potency overall based on their markedly lower IC_50_ value.

#### 3.4.2 Anti-inflammatory/anti-arthritic activity

The inflammation process is highly interconnected, as free radicals that harm cells can cause an inflammatory response. It is a reaction that serves as the defense mechanism of the body, eliminating undesired stimuli and triggering recovery. In addition, there is evidence that the stability of membranes is also helping in the anti-inflammatory effect of the extract. Ag@ZnO NCs (Fig. 8B) demonstrated the maximum protein denaturation at 1000 µg mL^−1^, reaching 83.6%, which is the most successful impact. It was found that Ag@ZnO NCs exhibited the lowest IC_50_ value of 39.28 µg mL^−1^, which is the lowest value, and it indicated that the formulation is the most efficient when it comes to protein denaturation. In the same concentration, diclofenac sodium produced denaturation 78.9% with an IC_50_ of 182.1 µg mL^−1^, and ZnONPs produced 78.8% of denaturation with an IC_50_ of 76.85 µg mL^−1^. AgNPs produced 74.9% protein denaturation at 1000 µg mL^−1^ and increased IC_50_ of 302.6 µg mL^−1^. These findings highlight the fact that 1000 µg mL^−1^ was the highest concentration at which the maximum protein denaturation was recorded in all groups, with Ag@ZnO NCs being the strongest, followed by ZnONPs and diclofenac sodium and AgNPs, based on their IC_50_ values.

### 3.5 Cytotoxicity analysis

#### 3.5.1 Antihaemolytic

**Fig. 9:**
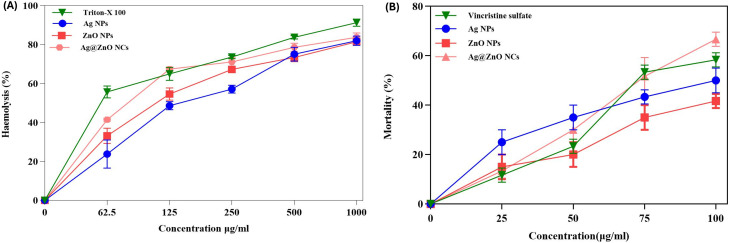
Cytotoxicity evaluation of synthesized nanoparticles: (A) haemolytic assay (B) brine shrimp lethality assay. Statistical significance from each group is shown by *p < 0.05.

The haemolytic activity of Triton-X 100, AgNPs, ZnONPs, and Ag@ZnO NCs increased in a concentration-dependent manner from 62.5 to 1000 µg mL^−1^ (Fig. 9A). Triton-X 100 exhibited the highest haemolysis, reaching approximately 92% at 1000 µg mL^−1^, confirming its strong membrane-disrupting effect as a positive control. Among the nanoparticles, Ag@ZnO NCs showed slightly higher haemolysis (84%) at 1000 µg mL^−1^ compared to ZnONPs (82%) and AgNPs (82%). The estimated IC_50_ values were approximately 60 µg mL^−1^ for Triton-X 100, ∼100 µg mL^−1^ for Ag@ZnONPs, ∼120 µg mL^−1^ for ZnONPs, and ∼200 µg mL^−1^ for AgNPs, indicating that AgNPs possess comparatively better hemocompatibility. Overall, the results demonstrate a clear dose-dependent haemolytic effect, likely due to nanoparticle interaction with red blood cell membranes, leading to increased permeability and haemoglobin release.

#### 3.5.2 Brine shrimp lethality assay

Lethality assay determines the toxicity of heavy metals, metal ions, nanoparticles, and plant bioactive compounds. The assay is common in both applied toxicology and research because it is simple, cheap, and has a small sample size. The biocompatibility of green-synthesized bimetallic NPs with the brine shrimp lethality assay has also been shown to guarantee their environmental and aquatic safety. The concentration of zinc oxide nanoparticles, ranging from 25 µg mL^−1^ to 100 µg mL^−1^, was the lethal concentration (LC_50_). The concentration of the nanoparticles was directly proportional to the level of lethality. All tested nanoparticles showed a clear concentration-dependent rise in mortality. AgNPs produced moderate toxicity, with mortality values increasing from 25% at 25 µg mL^−1^ to 55% at 100 µg mL^−1^, demonstrating a consistent linear progression in lethality (Fig. 9B). ZnONPs exhibited the weakest cytotoxic response, with mortality rising from 10–20% at 25 µg mL^−1^ to a maximum of 40% at 100 µg mL^−1^, indicating comparatively low bioactivity. In contrast, the Ag@ZnO NCs displayed markedly enhanced activity, recording 15% mortality at 25 µg mL^−1^ and reaching 65–70% at 100 µg mL^−1^, thus outperforming both individual nanoparticles across all concentrations. The standard drug vincristine sulphate showed expected dose-dependent lethality, ranging from 10% at 25 µg mL^−1^ to 55% at 100 µg mL^−1^. These findings demonstrate that the synergistic incorporation of Ag into the ZnO matrix substantially enhances lethality, leading to significantly higher cytotoxicity.

### 3.6 Antibacterial

**Fig. 10:**
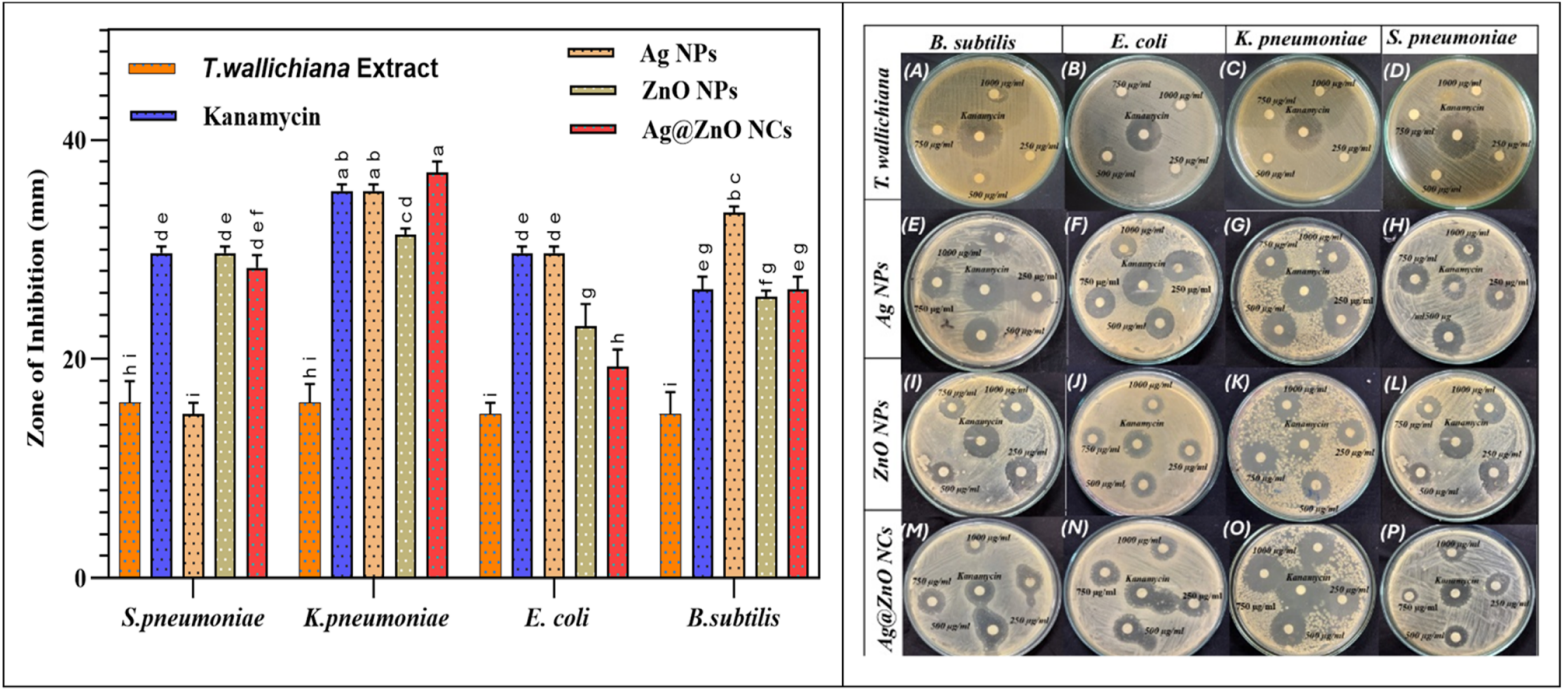
Zone of inhibition and antibacterial activity against the four mentioned strains; zones of inhibition produced by *T. wallichiana* extract (A–D), AgNPs (E–H), ZnONPs (I–L), and Ag@ZnO nanocomposites (M–P) against *B. subtilis*, *E. coli*, *K. pneumoniae*, and *S. pneumoniae*.

The *in vitro* antibacterial activities of Tw based synthesized compounds were determined against two Gram-negative (*E. coli* and *P. aeruginosa*) and two Gram-positive bacterial strains (*S. pyogenes* and *S. aureus*) by the disk diffusion method. The findings showed that all the tested compounds showed potent to moderate antibacterial activity with an inhibition zone of 6.00 ± 0.011 to 30.6 ± 0.283 mm (Fig. 10). Against *S. pneumoniae*, all samples exhibited moderate inhibition, with ZnONPs showing slightly higher activity (∼30 mm) compared with plant extract (∼17 mm), AgNPs (∼28 mm), and Ag@ZnO NCs (∼29 mm). The strongest antibacterial response was observed against *K. pneumoniae*, where Ag@ZnO NCs demonstrated the highest inhibition zone (∼38 mm), surpassing the plant extract (∼16 mm), ZnONPs (∼34 mm) and AgNPs (∼31 mm), indicating a strong synergistic effect of the composite material.

In contrast, *E. coli* was the least susceptible strain overall, showing reduced inhibition particularly with plant extract (∼15 mm), Ag@ZnO NCs (∼20 mm), and AgNPs (∼22 mm), while ZnONPs (∼29 mm). In general, the comparative analysis has revealed that Ag@ZnO NCs have the strongest antibacterial activity, particularly against *K. pneumoniae*, and ZnONPs demonstrate the highest activity against Gram-positive strains, in particular, *B. subtilis*. In the meantime, *E. coli* is most resistant to all the nanomaterials that are tested. These results underscore the fact that antibacterial activity is determined by the composition of nanoparticles and the structure of bacterial cell walls.

### 3.7 Phytotoxicity

To test whether the effects we observed on the seedlings carried over into improved growth, we examined growth in Petri plates. Shoot length and root length, along with germination (%), were recorded at different time intervals of all treated seeds. At all concentrations, the three nanoparticles (AgNPs, ZnONPs, and Ag@ZnO NCs) stimulated radish seedling growth in a dose-dependent manner. All NPs improved root length, shoot length, and germination (%) compared to the control. In terms of root length, Ag@ZnO NCs exhibited the largest increase, especially at higher concentrations (300–400 µg mL^−1^), where root length reached about 5–5.2 cm. ZnONPs were closely followed by AgNPs, which consistently produced the shortest roots among the nanoparticle groups while remaining larger than the control. In terms of shoot length at the highest concentration, 400 µg mL^−1^, Ag@ZnO NCs resulted in the greatest shoot elongation, reaching around 5 cm. ZnONPs had a somewhat lower effect with less shoot elongation, while AgNPs showed the least shoot enhancement among all concentrations of treated nanoparticles (Fig. S4A–C).

In terms of germination percentage, Ag@ZnO NCs demonstrated the largest improvement, reaching 85% germination rate at the highest concentration, whereas ZnONPs obtained around 80% germination, and AgNPs slightly lower. Germination was reduced for all NPs at lower concentrations (100–200 µg mL^−1^) compared to the control, but considerably enhanced at 300–400 µg. Overall, ZnONPs showed moderate enhancement, AgNPs showed the mildest but still discernible growth-promoting influence, and Ag@ZnO NCs showed the most significant positive impact on germination and root and shoot development, suggesting a synergistic effect of the bimetallic composition.

### 3.8 Principal component analysis and correlation

**Fig. 11:**
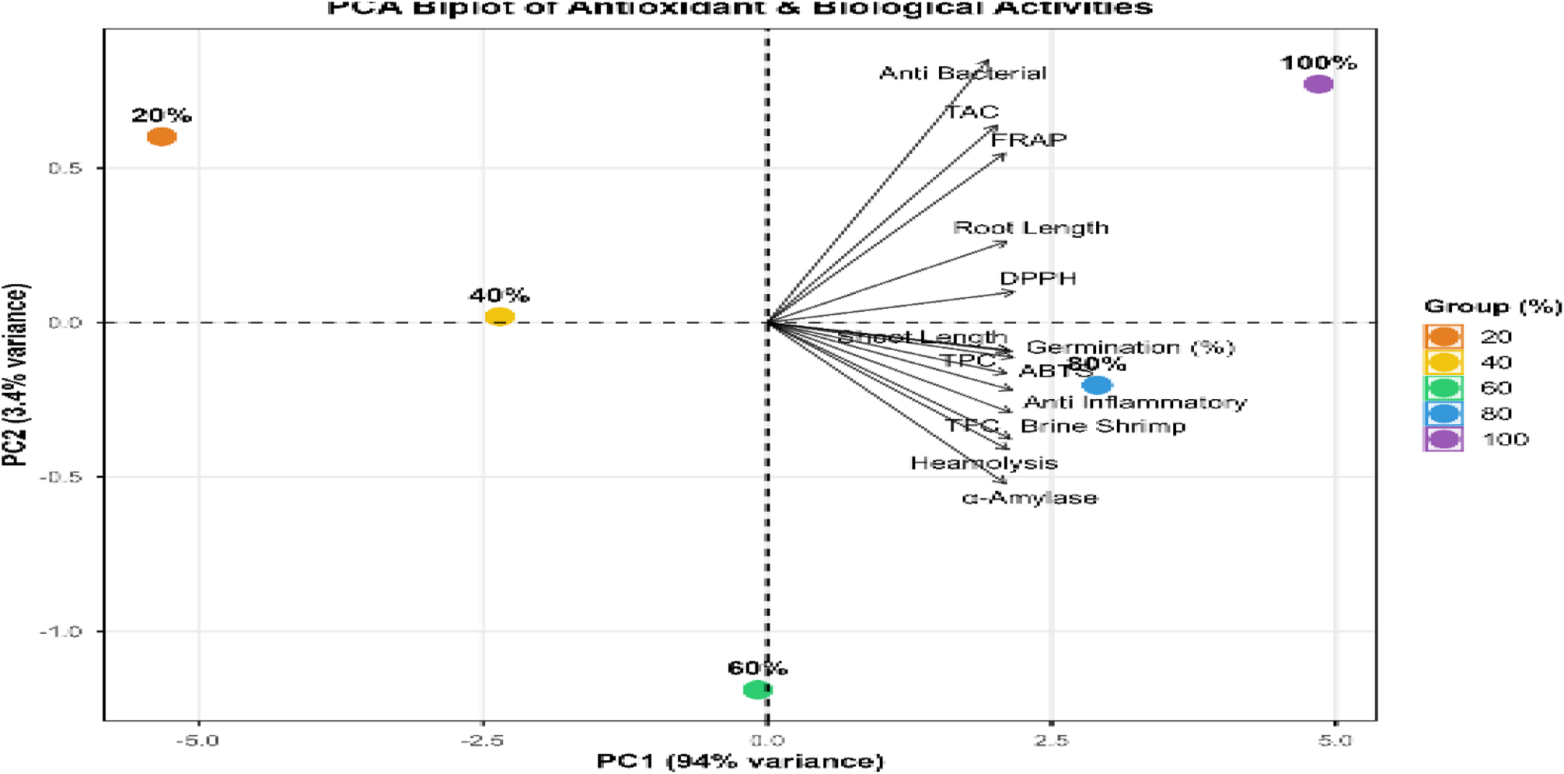
PCA biplot illustrating the clustering of samples (20–100%) and the contribution of antioxidant and biological activity variables to total variance (PC1 = 94%, PC2 = 3.4%).

The principal component analysis (PCA) biplot (Fig. S11) showed that 94% of the variance was explained by PC1 and 3.4% by PC2, giving a total variance of about 97.4%. The high cumulative variance value suggests that the biplot of the 2 dimensions is a good representation of the data set. PC1 represents the dominant component of the variance, with most variation attributed to antioxidant and phytochemical properties. As shown by the closely aligned vectors of all the major antioxidant and bioactivity variables (TAC, TPC, TFC, FRAP, DPPH, ABTS, anti-inflammatory, anti-amylase, and antibacterial activity), all the variables were strongly and positively correlated along PC1, which reflects a major antioxidant–bioactivity gradient. However, shoot and root length had an inverse or less pronounced relationship with antioxidant potential and were signified along PC1, thus suggesting the presence of phytotoxic effects. The distribution of the samples along PC1 also indicated a clear pattern of concentration dependence, with samples at higher treatments (*e.g.*, 100%) on the positive side of PC1 and correlated with higher antioxidant and biological activity, while lower treatments (*e.g.*, 20%) were on the negative side, indicating lower activity. Intermediate treatments (40–80%) fell somewhere between these extremes, showing that there were progressive changes in bioactivity. PC2, while accounting for a small percentage of the overall variance (3.4), did contribute a small but meaningful amount of variance, particularly in relation to haemolysis stability and antibacterial response, and this resulted in some vertical separation of samples on the biplot. Overall, the PCA results showed that antioxidant and functional biological properties are the most significant sources of variation, whereas the phytotoxicity-related properties have a secondary and opposing pattern (Fig. 11).

## 4 Discussion

The current study was intended to prepare and profile Ag, ZnO, and Ag@ZnO NCs with the *T. wallichiana* extract and assess their biological and functional features. The leaves were used for nanoparticle synthesis because of higher concentrations of flavonoids, phenolics, and diterpenoids, which act as reducing and stabilizing agents, whereas the bark and branches were not used in this study because they are pharmacologically active. The formation of precipitation when the NaOH is added is the nucleation stage of the synthesis of nanoparticles. The hydroxide ions in solution promotes reduction and the formation of oxides, which results in the appearance of precipitates. Certain phytochemicals present in *T. wallichiana* extract, such as flavonoids, phenolics, and proteins, however, bind to the surfaces of the particles and serve as capping agents that can inhibit uncontrolled aggregation. Ag and ZnO nanoparticles' bio applications are relatively new compared to traditional biomaterials used in recent years, particularly in developing countries. Notably, the Ag@ZnO NCs exhibited enhanced performance compared to their monometallic counterparts, indicating strong synergistic electronic interactions at the Ag@ZnO interface. Electron hole pairs are generated by photoexcitation in ZnO nanoparticles; these charge carriers limit the biological efficiency, which restricts ROS generation. Ag captures and stabilizes electrons by acting as an electronic sink and inhibits electron–hole recombination.^48^ These outcomes are consistent with earlier reports showing that plant-derived phytochemicals can effectively mediate nanoparticle formation while improving their biological activity.^49^

Monometallic AgNPs, ZnONPs, and bimetallic Ag@ZnO NCs were synthesized by a one-step method at room temperature utilizing aqueous leaf extract of *T*. *wallichiana* Linn. In each set of reactions, the color was not the same as one another based on the salt and concentration used. The reaction mixture for ZnO nanoparticles exhibited a light brown color, while the reaction mixture for AgNPs exhibited a grayish black color. In the case of bimetallic Ag@ZnO NCs synthesis, the color of the reaction mixture is dark brownish.^50^

TEM morphological analysis provided direct, high-magnification visual insights into the true physical boundaries of the individual nanodomains, completely separating individual particle traits from dry-state aggregation. By utilizing high-resolution imaging coupled with ImageJ analysis software, the individual physical primary particle diameters were explicitly tracked and measured, revealing ultra-fine nanoscale dimensions (4.94 ± 2.22 nm for AgNPs, 15.64 ± 4.69 nm for ZnONPs, and 6.42 ± 1.98 nm for Ag@ZnO NCs). These values show that the AgNPs are composed of distinct, small spherical nanodomains embedded within the matrix, in excellent agreement with recent high-surface-energy configurations.^51^ The Wurtzite crystal morphology of the ZnONPs is manifested by the presence of larger polycrystalline domains, while the bimetallic Ag@ZnO NCs exhibit a highly uniform distribution of compact domains indicating successful interfacial integration.^52^ Crucially, the presence of sharp, concentric SAED rings with bright spots across all three samples provides unambiguous proof of the highly ordered polycrystalline nature of these individual physical particles, eliminating any possibility of amorphous plant-mass interference.^53^

The Ag@ZnO NCs displayed an absorption peak at 390 nm, which is red-shifted relative to ZnONPs (384 nm),^54^ but blue-shifted compared to AgNPs (420 nm).^55–57^ This intermediate red shift indicates strong electronic interaction between Ag and ZnO at the interface. When Ag is coupled with ZnO, the plasmon resonance of Ag experiences a shift because the dielectric environment, band alignment, and charge-transfer dynamics are altered, producing a hybrid plasmon–exciton band instead of two separate peaks. Similar red-shifts are widely reported for Ag@ZnO heterojunctions, confirming successful nanocomposite formation.^58–60^ It is critical to note that the raw concentration of the reaction mixtures resulted in maximum absorbance intensities approaching or exceeding 3.0, which goes beyond the standard linear threshold of the Beer–Lambert law where light transmittance falls below 0.1%. Consequently, to ensure scientific accuracy, these electronic spectra are interpreted strictly from a qualitative perspective. They are utilized exclusively to confirm the successful completion of the synthesis reactions, identify characteristic Surface Plasmon Resonance (SPR) positions, and monitor relative band shifts, rather than for absolute quantitative concentration or absorbance metrics.

In X-ray diffraction, a mixture of both salts in the reaction mixture resulted in a change of size in nanoparticles depending on the salt used. An increase in the size of nanoparticles was observed upon the addition of ZnO salt at a higher concentration than silver salt. This has been observed by numerous researchers that silver itself or the addition of Ag salt increases the size of NPs compared to ZnO NP alone, or when added to the medium.^50^ AgNPs displayed planes of fcc silver.^57^ The ZnONPs planes of hexagonal wurtzite,^57^ ZnO, confirming good crystallinity.^61^ In the Ag@ZnO bimetallic nanocomposites, the major Ag peaks reappeared, while overlapping contributions from ZnO were also evident, indicating the coexistence of both crystal phases and successful formation of the composite structure.^58^ The slight broadening and intensity variation in the composite pattern suggest nanoscale interactions between Ag and ZnO domains.^58–60^ It is critical to clarify the methodological distinction between our solid-state structural characterizations: while powder XRD exclusively mathematically isolates the internal crystalline grain domain size of the cores *via* Scherrer calculations, our ImageJ-assisted TEM analysis evaluates the total physical dimensions of the individual capped nanoparticles. XRD analysis confirmed the formation of metallic Ag and crystalline ZnO phases, although silver nitrate and zinc acetate were used as precursor salts; thus, the synthesized nanomaterials were designated as AgNPs and ZnO NPs, respectively.

To understand the surface passivation and assembly mechanisms, FTIR bands (O–H/N–H, C=O/C=C, C–O) were systematically compared against the newly integrated spectrum of the pristine *T. wallichiana* extract. The direct tracking of the plant extract fingerprints across all samples definitively establishes that polyphenols, flavonoids, and proteinaceous metabolites actively serve as the primary capping and reducing ligands. These surface-bound biomolecules modulate the physical particle boundaries and directly dictate their long-range assembly behavior.^60,62^ To evaluate this bulk powder architecture, wide-field SEM surveys were performed at a unified 200× magnification with a 100 µm scale bar (Fig. 5). Instead of isolated individual particles, the long-range surveys revealed a highly textured, multi-layered macro-cluster morphology. AgNPs displayed rough, rock-like granular blocks, ZnONPs exhibited dense, plate-like agglomerated structures, and the bimetallic Ag@ZnO NCs revealed a highly complex, heterogeneous mixed architectural network where irregular granular domains are distributed over larger ZnO-like aggregates.^63^ This surface texturing and complex multi-layered aggregation are expected outcomes of green fabrication. The organic capping layer provided by the *T. wallichiana* extract provides vital functional groups for applications but concurrently screens the core electrostatic charges, promoting the primary nanostructures to assemble into stable micrometer-scale macro-clusters during drying and powder preparation. The EDX spectrum of AgNPs exhibits a great Ag signal (∼46.5 wt%), which supports the creation of the elemental silver, and the presence of the C and O signals indicates the presence of the phytochemical capping characteristic of the green synthesized nanoparticles. The fact that the dominant peaks in ZnONPs are Zn (53.1 wt%) and O proves the wurtzite ZnO presence, which is in line with the earlier reported biosynthesized ZnO profiles. The Ag@ZnO nanocomposites show both Zn (41.7 wt%) and Ag (31.1 wt%) signals in the same spectrum, and this is evidence of successful incorporation of both metals into one composite structure. Similar dual-element EDX signatures are widely reported for Ag@ZnO heterojunctions, supporting true nanocomposite formation rather than a physical mixture.^59,62^ The precursor ratio used in the synthesis was 1/10 (Ag/Zn). However, as shown in the EDX analysis in Fig. 6, the elemental composition of the final nanocomposite can be different from the initial precursor ratio due to the differences in reduction efficiency, nucleation, and incorporation of ions. The final composition approached 1/1. This was confirmed by EDX, which showed the preferential incorporation of silver in the ZnO matrix.

The solution-state DLS and zeta potential measurements provide perfect physical chemistry synchronization with our solid-state SEM/TEM images, cleanly resolving any apparent structural contradictions. Solution-state evaluations generated *Z*-average hydrodynamic diameters of 422.8 nm (PDI = 0.757) for AgNPs, 377.2 nm (PDI = 0.416) for ZnONPs, and a highly compact 195.6 nm (PDI = 0.985) for the bimetallic Ag@ZnO NCs (Fig. S1). Surface charge characterization revealed zeta potential values of −18.3 mV for AgNPs, −16.8 mV for ZnONPs, and an enhanced negative potential of −23.0 mV for the Ag@ZnO NCs. Thermodynamically, absolute surface charges below the classic 40 mV threshold confirm weak electrostatic repulsion and limited dispersion stability in suspension. This fundamental property explains why the solution-state DLS profiles track the collective hydrodynamic volumes of biomolecularly bound clusters rather than isolated individual cores. Rather than contradicting our nanoscale claims, this indicates that while individual primary physical cores are kept strictly at the nanometer scale (4–15 nm) during initial nucleation *via* plant capping, their low surface charges cause them to naturally assemble into the stable, large-scale macro-clusters captured in the wide-field SEM surveys. Crucially, the bimetallic Ag@ZnO heterojunction yields a higher surface potential (−23.0 mV), enhancing charge-based repulsion and suppressing continuous overgrowth, which mathematically explains why the bimetallic system maintains a significantly reduced hydrodynamic volume compared to its monometallic counterparts.^54,59,64^

The TFC graph shows that the plant extract contains the highest flavonoid content at all concentrations, followed by Ag@ZnO NCs, while ZnONPs and AgNPs have comparatively lower TFC values. In contrast, the TAC/TPC-related graph indicates that ascorbic acid exhibits the strongest antioxidant capacity, whereas among nanoparticles, AgNPs show the highest activity, followed closely by Ag@ZnO NCs, with ZnONPs showing the least antioxidant response. Overall, the plant extract is richest in flavonoids, but AgNPs and Ag@ZnO NCs demonstrate superior antioxidant performance compared to ZnONPs, reflecting differences in redox behaviour rather than flavonoid content.^65,66^ To systematically establish the therapeutic advantage of this green process, the biological assays explicitly evaluated the crude, metal-free *T*. *wallichiana* extract alongside the biofabricated materials. The outcomes show that while the standalone matrix possesses inherent baseline medicinal properties, the green synthesis route significantly amplifies and complements this baseline efficacy.

Good antioxidant activity, while moderate enzyme inhibition is observed. While a direct experimental comparison with conventionally/chemically synthesized bare nanoparticles was outside the eco-friendly scope of this green chemistry investigation, our findings were juxtaposed against established literature values for bare chemical equivalents. This comparison highlights that our plant-mediated nano-architectures exhibit a superior biological profile. This enhancement is directly dictated by the bioactive phytochemical ligands passivating the nanoparticle surface, which work in tandem with the core elements to drive a highly cooperative, synergistic therapeutic response. Ag@ZnO NCs showed the highest antioxidant activity due to synergistic charge-transfer interactions between Ag and ZnO, which enhance electron-donation and radical-scavenging ability, consistent with reports of improved redox performance in Ag@ZnO NCs bimetallic systems.^62^ Separation of charges improves ROS production, thereby enhancing antioxidant and antimicrobial activity. Previous studies have demonstrated that Ag-decorated ZnO systems generate substantially higher ROS levels than ZnO alone, owing to electron trapping at the heterojunction interface. Cellular damage, oxidative stress, and membrane disruption caused by an increase in ROS production lead to enhanced antibacterial efficacy.^67^ Ag@ZnO NCs indicated slightly higher antimicrobial potential than ZnONPs. The antimicrobial performance was stable when nanoparticles were formed using nanocosmeceuticals.^54,58,65^ Nanoparticles produced by bioengineering showed promising biological characteristics and thus require further investigation to ascertain their pharmacological importance.^59,68^

The anti-inflammatory assay shows that Ag@ZnO NCs possess the highest inhibition of protein denaturation across all concentrations, reflected by the lowest IC_50_ value, indicating superior anti-inflammatory potential compared to the monometallic nanoparticles. ZnONPs exhibit moderate activity, while Ag NPs display the weakest inhibitory effect with the highest IC_50_. At elevated concentrations, Ag@ZnO NCs even surpass the reference drug, demonstrating the strongest overall anti-inflammatory response among all tested formulations.^46,69,70^ The results of α-amylase activity indicate that Ag@ZnO NCs are the strongest inhibitor, evidenced by the highest activity and lowest IC_50_. AgNPs are moderately inhibited, and ZnONPs are the least inhibited. On the whole, the results show that Ag@ZnO NCs are superior in terms of α-amylase inhibitory capacity than the monometallic nanoparticles and even the standard drug.^71,72^ The brine shrimp assay shows a clear dose-dependent increase in lethality for all nanoparticles, with Ag@ZnO NCs exhibiting the highest cytotoxicity, indicating strong biological activity. AgNPs show moderate toxicity, while ZnONPs demonstrate the lowest lethality, suggesting comparatively better biocompatibility. Overall, the results conclude that Ag@ZnO NCs are the most cytotoxic, followed by AgNPs, whereas ZnONPs are the least toxic under the tested conditions.^71,73–75^ The haemolysis results show that AgNPs cause the least red-blood-cell damage, indicating the best hemocompatibility. ZnONPs produce moderate haemolysis, while Ag@ZnO NCs show slightly higher haemolysis than both monometallic nanoparticles. Overall, AgNPs are the safest, followed by ZnONPs, with Ag@ZnO NCs being the most haemolytic among the nanoparticle groups.^69,71,75,76^

## 5 Conclusion

This study adopted an eco-friendly approach to successfully synthesize AgNPs, ZnONPs, and bimetallic Ag@ZnO NCs utilizing the leaf extract from *T. wallichiana*. The successful fabrication of the bimetallic heterojunction was confirmed by characteristic UV-vis surface plasmon resonance transitions. While X-ray diffraction mathematically isolated internal crystallite grain structures, high-magnification TEM profiling coupled with ImageJ analysis software directly resolved the distinct physical boundaries of the individual primary nanoparticles, establishing their fine nanoscale dimensions. Concentric SAED patterns confirmed the highly ordered polycrystalline nature of these metallic domains. Concurrently, wide-field SEM surveys captured the long-range assembly of these primary structures into textured, multi-layered micrometer-scale macro-clusters, a behavior driven by phytochemical surface passivation and low absolute electrostatic surface charges as mapped by FTIR and liquid-phase zeta potential metrics. These unique structural arrangements and interfacial electronic configurations significantly enhanced the surface reactivity and multi-therapeutic performance of the materials. The bimetallic Ag@ZnO NCs demonstrated the most powerful biological efficiency, exhibiting significant antioxidant and anti-inflammatory properties that outperformed their monometallic counterparts. Furthermore, the bimetallic nanocomposites displayed remarkable antibacterial efficacy, creating substantial zones of inhibition against pathogenic bacterial lines.

Biocompatibility profiling against human erythrocytes (RBCs) and brine shrimp larvae indicated concentration-dependent mortality trends, suggesting that a degree of caution should be observed in direct systemic applications. Overall, the findings highlight that *T. wallichiana*-passivated Ag@ZnO NCs represent highly promising candidates for biomedical, environmental, and advanced agricultural applications, particularly in treating disorders governed by oxidative stress. Future *in vivo* trials are recommended to firmly establish safety windows and validate these therapeutic actions within complex biological systems, alongside detailed molecular investigations into the precise pathways driving their enzyme inhibition and anti-inflammatory responses.

## Ethics approval and consent to participate

All experiments involving human blood samples were performed in accordance with the Guidelines of the Declaration of Helsinki, and the experimental protocol was approved (Approval no. Bch#0256) by the Bioethical Committee (BEC) of Quaid-i-Azam University, Islamabad, Pakistan. Informed consents were obtained from all human participants involved in this study.

## Author contributions

Mehreen Sarfraz: conceptualization, visualization, data curation, investigation, methodology, software, and writing – original draft. Shahid Sultan: data curation, formal analysis, software, and writing – review & editing. Amjid Khan: methodology, data curation, characterizations, resources, validation, funding acquisition, and writing – review & editing. Umer Rehman and Shanzay Saleem: formal analysis, and writing – review & editing. Muhammad Ali: data curation, formal analysis, resources, validation, and writing – review & editing. Hamza Elsayed Ahmed Mohamed: formal analysis, resources and characterizations. Zabta Khan Shinwari: project administration, supervision, conceptualization, validation, and writing – review & editing. All authors read and approved the final manuscript.

## Conflicts of interest

The authors declare that they have no known competing financial interests or personal relationships that could have appeared to influence the work reported in this paper.

## Supplementary Material

RA-016-D6RA04018G-s001

## Data Availability

The data supporting this article are provided in supplementary information (SI). Supplementary information is available. See DOI: https://doi.org/10.1039/d6ra04018g.
