# Supplementary material for: Green synthesis and characterization of Ag and ZnO nanoparticles and Ag@ZnO nanocomposites using Taxus wallichiana: comparative assessment of therapeutic potential and biocompatibility
Source: RSC Adv. 2026 Jul 3;16(35):36717–36. doi: 10.1039/d6ra04018g (PMC13331685; doi:10.1039/d6ra04018g)
Supplement: RA-016-D6RA04018G-s001 [file RA-016-D6RA04018G-s001.pdf]

Supplementary Information (SI) for RSC Advances.
This journal is © The Royal Society of Chemistry 2026

Green Synthesis and Characterization of Ag and ZnO Nanoparticles and Ag@ZnO Nanocomposites using *Taxus wallichiana*: Comparative Assessment of Therapeutic Potential and Biocompatibility

Mehreen Sarfraz[1], Shahid Sultan[1], Amjid Khan[2,3*], Umer Rehman[1], Shanzay Saleem[4], Muhammad Ali[5,6], Hamza Elsayed Ahmed Mohamed[7], Zabta Khan Shinwari[1,8*]

[1]Department of Plant Sciences, Faculty of Biological Sciences, Quaid-i-Azam University, Islamabad, 45320, Pakistan

[2]UNESCO-UNISA Africa Chair in Nanosciences and Nanotechnologies, College of Graduate Studies, University of South Africa, 1 Preller Street, Muckleneuk Ridge, P.O. Box 392, Pretoria, Gauteng Province, 0003, South Africa

[3]African Centre of Competencies in Enhanced Nanosciences & Nanotechnologies for SDGs (ACCENTS), 1 Preller Street, Muckleuneuck Ridge, P.O. Box 392, Pretoria, Gauteng Province, 0003, South Africa

[4]Department of Botany, The University of Punjab, Lahore, 54590, Pakistan

[5]Department of Biotechnology, Faculty of Biological Sciences, Quaid-i-Azam University, Islamabad, 45320, Pakistan

[6]Dr. A Q. Khan Institute of Materials & Emerging Sciences – DAKIMES, Quaid-i-Azam University, Islamabad, 45320, Pakistan

[7]Physics Department, Institut des Molécules et Matériaux du Mans, Le Mans Université, Le Mans, France

[8]Federal Urdu University of Arts, Sciences and Technologies (FUUAST), Karachi, 75300, Pakistan

***Corresponding Author**

Amjid Khan (khana2@unisa.ac.za)

Zabta Khan Shinwari (shinwari@qau.edu.pk)

Supplementary Data

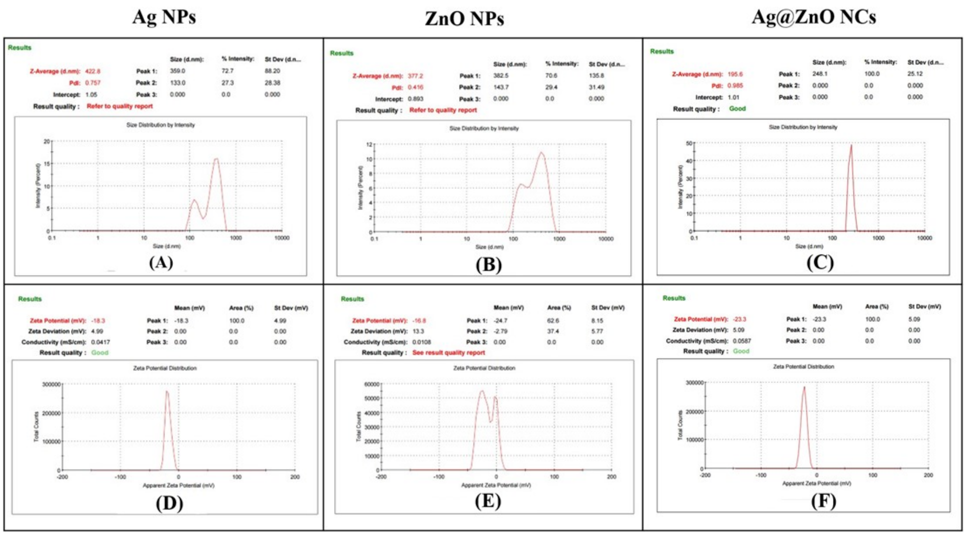

Figure S1. Illustration of DLS of AgNPs (A), ZnONPs (B), and Ag@ZnO NCs (C), and zeta potential of AgNPs (D), ZnO NPs(E), and Ag@ZnO NCs (F) of green-synthesized *T. wallichiana* extract-based nanoparticles.

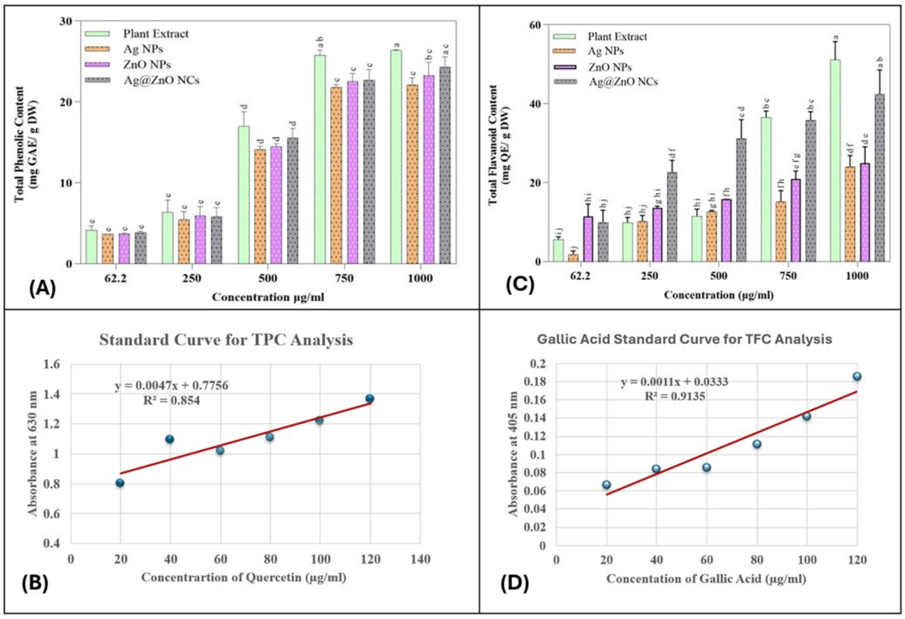

Figure S2. Phytochemical and standard curve analysis of *T. wallichiana* extract-based nanoparticles; (A, B) Total phenolic contents (TPC), (C, D) Total flavonoids content (TFC).

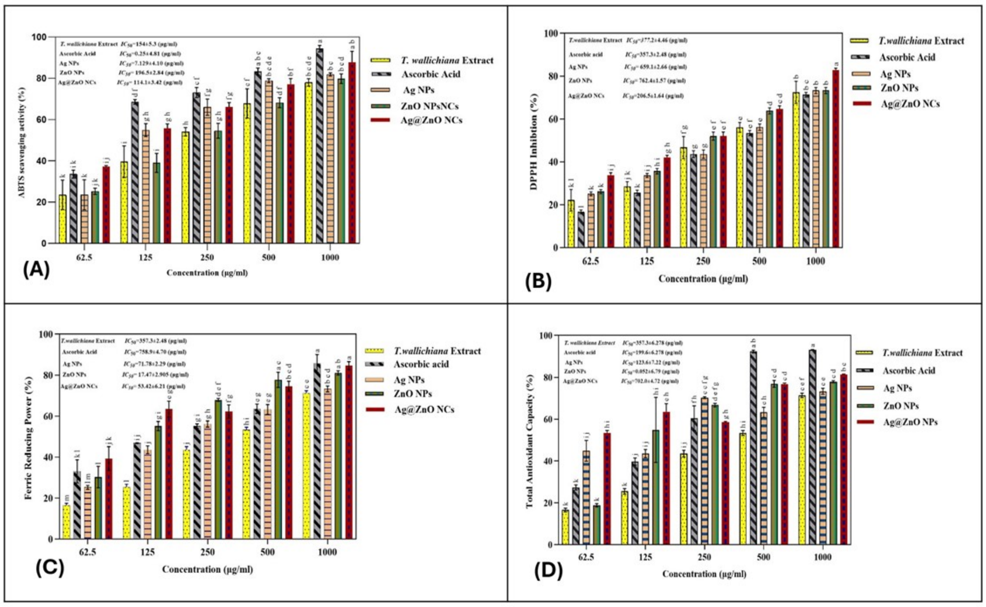

Figure S3. Antioxidant activities of *T. wallichiana* extract and *T. wallichiana*-based AgNPS, ZnONPs, and Ag@ ZnO NCs, (A) $ABTS^{\bullet+}$ Radical Scavenging Activity, (B) DPPH assay, (C) Ferric Reducing Antioxidant Power (FRAP), and (D) Total Antioxidant Capacity (TAC).

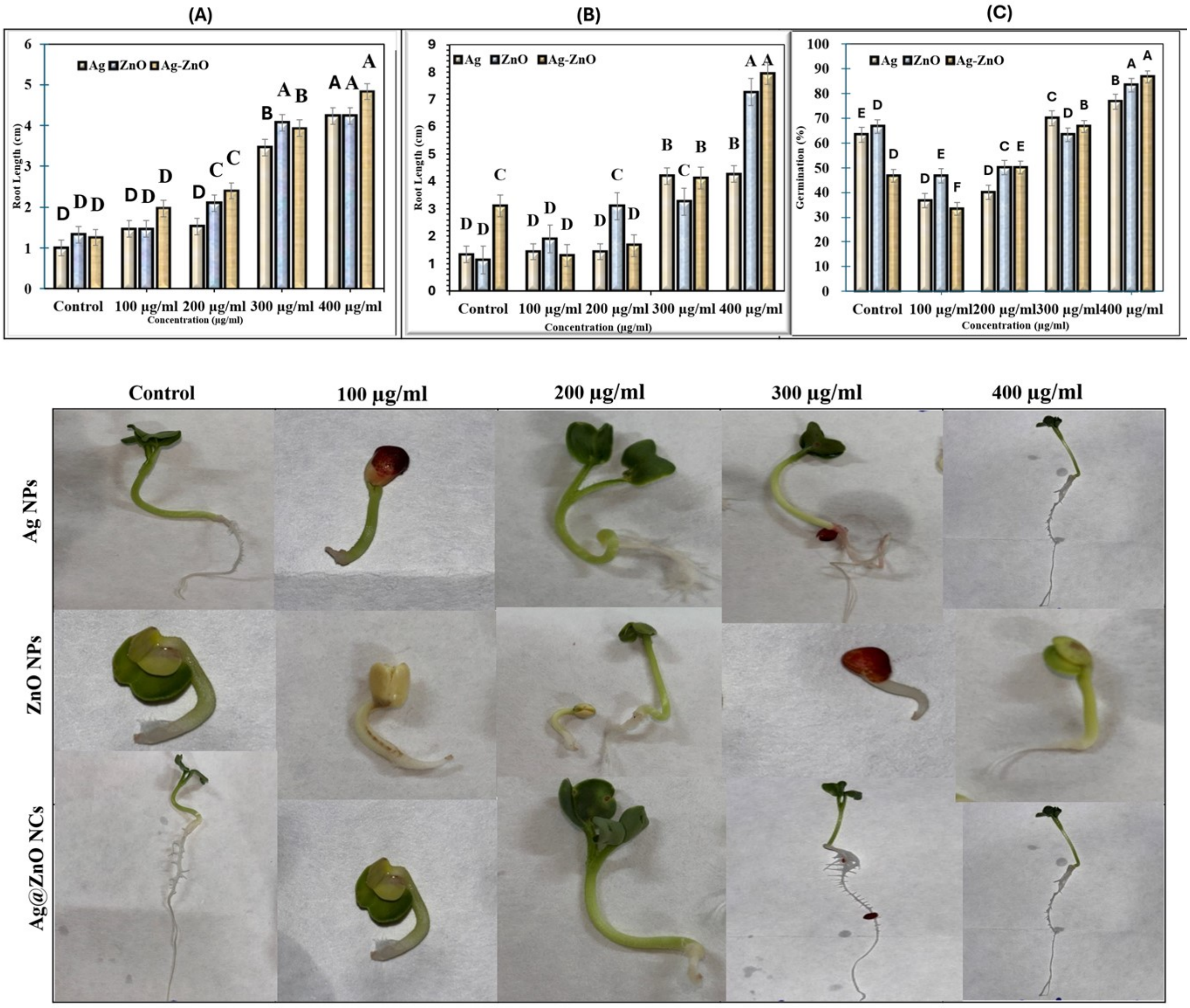

Figure S4. Illustration of the graph (A-C) Root length, shoot length, and germination percentage. Below are the images of radish seedlings treated with AgNPs, ZnONPs, and Ag@ZnO NCs at 100-400µg/ml, showing dose-dependent effects on shoot length, root length, and germination.

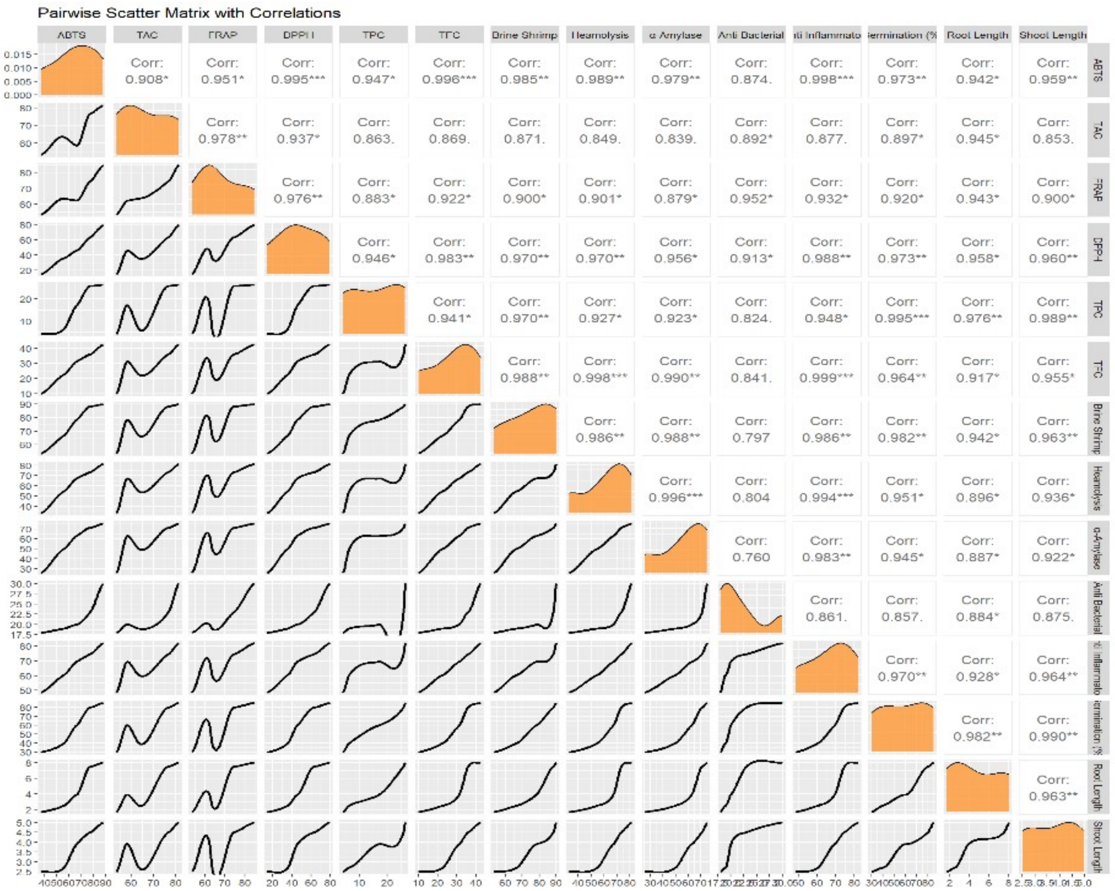

Figure S5. Pairwise correlation matrix showing relationships among antioxidant assays and biological activity parameters.
